# Dynamic changes in the proximitome of neutral sphingomyelinase-2 (nSMase2) in TNFα stimulated Jurkat cells

**DOI:** 10.3389/fimmu.2024.1435701

**Published:** 2024-07-09

**Authors:** Marie Schöl, Rebekka Schempp, Thomas Hennig, Dominik Wigger, Fabian Schumacher, Burkhard Kleuser, Christian Stigloher, Marco van Ham, Lothar Jänsch, Sibylle Schneider-Schaulies, Lars Dölken, Elita Avota

**Affiliations:** ^1^ Institute for Virology and Immunobiology, University of Wuerzburg, Würzburg, Germany; ^2^ Institute of Pharmacy, Department of Pharmacology & Toxicology, Freie Universität Berlin, Berlin, Germany; ^3^ Imaging Core Facility, Biocenter, University of Wuerzburg, Würzburg, Germany; ^4^ Cellular Proteome Research Group, Helmholtz Centre for Infection Research, Braunschweig, Germany; ^5^ Institute of Virology, Medizinische Hochschule Hannover, Hannover, Germany

**Keywords:** neutral sphingomyelinase 2 (nSMase2), tumor necrosis factor-alpha (TNF-alpha), proximity labeling, APEX2, proteomics, ceramide, plasma membrane (PM), protein dynamic

## Abstract

Ceramides generated by the activity of the neutral sphingomyelinase 2 (nSMase2) play a pivotal role in stress responses in mammalian cells. Dysregulation of sphingolipid metabolism has been implicated in numerous inflammation-related pathologies. However, its influence on inflammatory cytokine-induced signaling is yet incompletely understood. Here, we used proximity labeling to explore the plasma membrane proximal protein network of nSMase2 and TNFα-induced changes thereof. We established Jurkat cells stably expressing nSMase2 C-terminally fused to the engineered ascorbate peroxidase 2 (APEX2). Removal of excess biotin phenol substantially improved streptavidin-based affinity purification of biotinylated proteins. Using our optimized protocol, we determined nSMase2-proximal biotinylated proteins and their changes within the first 5 min of TNFα stimulation by quantitative mass spectrometry. We observed significant dynamic changes in the nSMase2 microenvironment in response to TNFα stimulation consistent with rapid remodeling of protein networks. Our data confirmed known nSMase2 interactors and revealed that the recruitment of most proteins depended on nSMase2 enzymatic activity. We measured significant enrichment of proteins related to vesicle-mediated transport, including proteins of recycling endosomes, trans-Golgi network, and exocytic vesicles in the proximitome of enzymatically active nSMase2 within the first minutes of TNFα stimulation. Hence, the nSMase2 proximal network and its TNFα-induced changes provide a valuable resource for further investigations into the involvement of nSMase2 in the early signaling pathways triggered by TNFα.

## 1 Introduction

Neutral sphingomyelinase 2 (nSMase2), encoded by the phosphodiesterase 3 (*SMPD3*) gene, is a key enzyme in the sphingolipid metabolism which catalyzes the conversion of sphingomyelin into ceramide (1–3). nSMase2 relies on Mg^2+^, anionic phospholipids such as phosphatidylserine, and a neutral pH for optimal sphingomyelin turnover (4–6). Typically, nSMase2 localizes to the cytosolic leaflet of the plasma membrane (PM), but localization to the Golgi apparatus has also been described (7, 8). Stress-induced ceramide generation mediated by nSMase2 has been reported in various processes, including inflammation and apoptosis, and, at a systemic level, heart failure, bone mineralization, or Alzheimer`s disease (9–14). Over the past two decades, nSMase2 activation has been shown to play an important role in the response to the inflammatory cytokine tumor necrosis factor α (TNFα) (15–19). In A549 epithelial cells, p38-α mitogen-activated protein kinase (MAPK (MAPK14)) and protein kinase Cδ were identified as nSMase2 upstream components of the TNF receptor 1 (TNFR1) promoting cell adhesion and migration (20, 21). TNFα-dependent nSMase2 activation promotes the signaling of MAPK and NFκB pathways, resulting in enhanced cytokine production in primary monocytic/macrophage cells (22). Similarly, exosome secretion in response to TNFα is positively affected by nSMase2 and ceramide-dependent modulation of vacuolar H^+^-ATPase activity (23). A neutral sphingomyelinase activation domain (NSD) within the TNFR1 serves as the binding site for FAN (factor associated with nSMase2 activation) (24). In Jurkat cells, FAN subsequently recruits EED (polycomb protein embryonic ectoderm development) and RACK1 (receptor for activated C-kinase 1) to form an nSMase2 signaling complex (25–27). Although nSMase2 is activated within the first minutes after TNFR engagement, the role of nSMase2 in the early signaling of TNFα is insufficiently studied.

Classical methods such as yeast-two-hybrid and immunoprecipitation-based mass spectrometry (MS) analysis have been employed to identify nSMase2-interacting proteins and to elucidate their role in TNFα signaling (20, 27, 28). However, these approaches have limitations in capturing rapid changes of weak and transient interactions, e.g., during the first few minutes of TNFα exposure. To overcome these challenges, proximity-dependent labeling techniques in living cells, coupled with affinity purification, have emerged as an excellent tool to capture and resolve protein networks (29–32). If genetically tagged with the engineered peroxidase APEX2, proteins of interest and their proximitome can be rapidly biotinylated in living cells pre-loaded with biotin-phenol (BP) upon adding hydrogen peroxide (H_2_O_2_). The resulting phenoxyl radicals promote the biotinylation of cellular proteins at electron-rich amino acid residues, such as tyrosine, within a radius of about 20 nm (33). Biotinylated proteins are then purified using streptavidin beads followed by mass spectrometry (MS) (34). This technique offers high temporal resolution and has proven effective in unraveling intricate interaction networks of receptors such as agonist-activated G-protein coupled receptors or antigen-induced changes after B-cell receptor activation (31, 35–38).

To explore the PM proximal network of nSMase2 and TNFα-induced changes thereof, we generated Jurkat cells stably expressing the nSMase2-APEX2 fusion protein. First, we tried to validate the APEX2 approach by proteomic analysis of nSMase2 proximal proteins under steady-state conditions. Recently, we published the lipid droplet protein PLIN3 as one of the most highly enriched proteins in the proximity of the nSMase2-APEX2 (39). We confirmed that by showing that PLIN3 and nSMase2 are co-detected in lipid droplets. The result indicated the suitability of our approach to resolve the changes in the nSMase2 proximal protein network upon TNFα stimulation. Importantly, we improved the capture efficiency of biotinylated proteins by removing excess biotin through size-exclusion centrifugation, allowing us to detect transient and dynamic protein complex formation within the initial five minutes of TNFα stimulation. We conducted an in-depth analysis of the evolving proximal proteome of nSMase2-APEX2 in Jurkat cells, revealing dynamic changes upon TNFα stimulation, with the majority dependent on nSMase2 activity. Subsequently, functional annotation of proteins revealed components of membrane trafficking and rearrangement of vesicle-mediated transport in TNFα stimulated cells, as reflected by the enrichment of proteins associated with recycling endosomes and exocytic vesicles in the nSMase2 proximitome. In conclusion, the current study provides a novel data set of nSMase2 proximal proteins that are enriched early after TNFα stimulation.

## 2 Materials and methods

### 2.1 Generation of stable Jurkat cell lines and cell cultivation

pcDNA3.1-based NSM2 (nSMase2)-GFP and H639A-GFP expression vectors were provided by V. Kozjak-Pavlovic (Institute of Microbiology, University of Würzburg). cDNA of NSM2 and its mutant version H639A were cloned into pWPI-EGFP-FLAG-APEX2 lentiviral vector (provided by G. Gerold, University of Veterinary Medicine Hannover) by In-Fusion cloning under the promotor EF-1α for optimal expression in Jurkat cells. A Gly-/Ser-rich linker sequence was inserted between NSM2 and the C-terminal EGFP-FLAG-APEX2. Lentiviral expression vectors of nSMase2 fusion proteins were used to generate lentivirus particles containing supernatants by transfection of HEK293 cells and subsequent transduction of Jurkat cells. EGFP-positive cells were selected by Flow Cytometry-based Cell Sorting (FACS) after transduction. Polyclonal cell lines bearing stably integrated expression constructs were selected and maintained in 10% fetal calf serum (FCS) Roswell Park Memorial Institute (RPMI) medium supplemented with blasticidin at a final 8 μg/mL concentration. The cell lines present in this study were obtained from ATCC (Jurkat clone E6–1) and BioCat GmbH (HEK293TN).

### 2.2 Thin-layer chromatography based NSM activity assay

NSM activity in Jurkat cells was measured as described previously (40). Briefly, Jurkat cells were lysed by freeze/thawing (4x, liquid nitrogen) in 25 mM Tris-HCl (pH 7.4), 0.1 mM phenylmethylsulfonyl fluoride (PMSF) and Protease Inhibitor Tablet (Thermo Fisher Scientific). 100 μg (endogenous NSM activity) or 20 μg (nSMase2 overexpression) of total protein was mixed with 50 mM Tris HCl (pH 7.4), 10 mM MgCl_2_, 0.2% Triton X-100, 10 mM DTT, 50 μM phosphatidylserine (Sigma-Aldrich, Germany), and 0.6 μM BODIPY-FL-C12 Sphingomyelin (N-(4,4-difluoro-5,7-dimethyl-4-bora-3a,4a-diaza-s-indacene-3-dodecanoyl) sphingosyl phosphocholine) (Thermo Fisher Scientific) in a total volume of 100 μL, and incubated at 37°C for 1 h. Reactions were stopped by MeOH: CHCl_3_ (2:1) and subjected to a Bligh and Dyer lipid extraction (41). Lipid extracts were spotted onto a TLC plate (Macherey-Nagel) and developed in MeOH: CHCl_3_ (80:20). The plate was air-dried, and lipid bands were visualized and quantified using Odyssey Fc Imaging System (LI-COR Biosciences).

### 2.3 Live-cell microscopy

Cells stably expressing nSMase2-APEX2 or H639A-APEX2 were transferred into µ-slide 18 well slides (Ibidi) and immediately imaged by confocal laser scanning microscopy. Images were acquired through the 488 nm filter channel at room temperature (RT) using an LSM 510 Meta (Zeiss, Germany), and image acquisition was performed with Zeiss LSM software 3.2 SP2.

### 2.4 Electron microscopy

The electron microscopy samples were prepared according to a previously published protocol (42). Transmission electron microscopy was performed at 120 kV acceleration voltage at a JEM-1400 Flash (JEOL, Germany) transmission electron microscope equipped with a Matataki 2k x 2k camera.

### 2.5 Flow cytometric analysis of TNFR1 internalization

Flow cytometric analysis of receptor surface immunofluorescence was used to determine agonist-induced internalization. Jurkat cells stably expressing nSMase2-APEX2 or H639A-APEX2 were incubated with TNF-R1 antibody (1:100, Santa Cruz Biotechnology) on ice for 20 min, washed with FACS buffer (0.4% BSA, 0.01% NaN_3_ in PBS) and either left unstimulated or incubated with 100 ng/mL TNFα (Thermo Fisher Scientific) for the noted time (0–15 min) at 37°C. All cells were washed and subsequently stained with Alexa647 goat anti-mouse (1:200; Invitrogen) for 30 min on ice. The median fluorescence intensity (MFI) of 10,000 cells per condition was measured using a FACSCalibur instrument (Becton Dickinson).

### 2.6 Quantification of phosphorylated proteins

For analysis of phosphorylated proteins, 1x 10^6^ Jurkat cells expressing nSMase2-APEX2 or H639A-APEX2 fusion protein were stimulated with 100 ng/mL TNFα in 200 μL 10% RPMI for 0, 1, 2, and 5 min. The reaction was stopped by immediately transferring the cells to ice and lysing in ice-cold RIPA buffer supplemented with a phosphatase inhibitor (Merck). Subsequently, phosphorylation was analyzed by WB.

### 2.7 Plasma membrane isolation

PM fractions were isolated from 2.5x10^7^ Jurkat cells utilizing Minute™ Plasma Membrane Protein Isolation and Cell Fractionation Kit (Invent Biotechnologies), following the manufacturer’s instructions with modifications in the lysis procedure. Specifically, Jurkat cells were lysed by passing them through a syringe with a 26GA needle eight times instead of using a filter cartridge.

### 2.8 Ceramide quantification by HPLC-MS/MS

The sphingolipid extraction was performed as previously described (43). Briefly, 1.5 mL MeOH: CHCl_3_ (2:1, v:v) was added to the sample (1x10^6^ cells; PM fractions) and was incubated overnight at 48°C. The internal standard C17:0 ceramide (C17:0 Cer, from Avanti Polar Lipids, Alabaster, USA), of which 2.5 pmol was finally injected into the column, was part of the extraction solvent. After addition of 150 µL KOH (1 M in MeOH) and incubation for 2 h at 37°C, 120 rpm, the lipid extract was neutralized with 12 µL glacial acetic acid and centrifugated (2,200 g, 5 min, 4°C). The organic phase was transferred to new extraction vessels and dried under vacuum. The following HPLC-MS/MS analysis was carried out under already published conditions (44). The lipid extract was chromatographically separated using a 1290 Infinity II HPLC (Agilent Technologies, Waldbronn, Germany) equipped with a Poroshell 120 EC-C8 column (3.0 × 150 mm, 2.7 µm; Agilent Technologies). Analysis in MS/MS mode was performed using a 6495C triple-quadrupole mass spectrometer (Agilent Technologies) in positive electrospray ionization mode (ESI+). Ceramide subspecies (C16:0, C18:0, C20:0, C22:0, C24:0 and C24:1) were quantified by multiple reaction monitoring (qualifier product ions in parentheses): [M-H_2_O+H]^+^ → *m/z* 264.3 (282.3). An external calibration was performed, and peak integration and analyte quantification were determined using MassHunter Quantitative Analysis Software (version 10.1, Agilent Technologies).

### 2.9 Western blot analysis

15 µg protein of whole cell lysates were subjected to SDS-PAGE. Antibody specific for FLAG (1:1000) was purchased from Merck, and GAPDH (1:1000) was purchased from Santa Cruz. Phospho-p38 (Th180/Tyr182) (1:1000), phospho-NFκB p65 (Ser536) (1:1000) and phospho-SAPK/JNK (Thr183/Tyr185) (1:1000) were from Cell Signaling. The bands were visualized using SuperSignal West Pico PLUS detection reagent (Thermo Fisher Scientific), and quantification was performed using the Odyssey Fc Imaging System (LI-COR Biosciences).

### 2.10 APEX2-mediated proximity labeling

APEX2 proximity labeling was performed according to the published protocol from Hung et al. with optimization for Jurkat cells (34). Briefly, 1.5x10^7^ Jurkat cells stably expressing nSMase2-APEX2 were incubated in 30 mL 10% RPMI containing 1 mM BP (Iris Biotech) at 37°C on a rotary wheel for 30 min.; cells were pelleted for 5 min at 457 g and resuspended in 3 mL 10% RPMI for stimulation and labeling reaction. 100 ng/mL TNFα (Thermo Fisher) was added, and cells were incubated at 37°C using a water bath for the indicated time points (1, 2, and 5 min). To initiate the labeling, H_2_O_2_ (30% (wt/wt), Sigma-Aldrich) was added to a final concentration of 1 mM for the final 1 min of stimulation. For the respective negative control, labeling was initiated as described, but TNFα was omitted. The APEX2 labeling reaction was quenched by the addition of 20 mL ice-cold quenching solution (10 mM ascorbate, 5 mM Trolox, and 10 mM sodium azide in PBS) to the cell suspension. Cells were pelleted immediately (1 min at 2862 g, 4°C), and the supernatant was discarded. Cells were washed 3x with a quenching solution and resuspended in 1.5 mL of ice-cold PBS. 5x10^6^ cells (~ 500 μL) were separated and used to validate TNFα signaling activation and successful biotinylation by western blot. The remaining sample was pelleted and lysed in 100 μL RIPA buffer (50 mM Tris-HCl (pH 7.5), 150 mM NaCl, 1% (vol/vol) Triton X-100, 0.1% SDS, 0.5% deoxycholate, 1 mM PMSF, Protease Inhibitor Cocktail (Thermo Fisher Scientific)) supplemented with quenchers (10 mM sodium azide, 10 mM sodium ascorbate and 5 mM Trolox) for 20 min on ice.

### 2.11 Streptavidin pull-down of biotinylated proteins

To ensure maximum recovery of biotinylated proteins, excess BP was removed by filtering the post-nuclear lysates using 3K MWCO protein concentrator columns (Pierce, Thermo Fisher Scientific). Biotinylated proteins were then enriched using 20 µL streptavidin magnetic beads (Pierce, Thermo Fisher Scientific) per sample at 4°C overnight. The bead-bound proteins were then washed with a series of buffers (2x RIPA, 1x 1 M KCl, 1x 0.1 M Na_2_CO_3_, 1x 2 M urea in 10 mM Tris-HCl, pH 8.0, 2x RIPA) and either subjected to down-stream MS or analyzed by western blot. For the latter, biotinylated proteins were eluted from the beads by boiling each sample in 40 μL of 3x protein loading buffer supplemented with 2 mM biotin and 20 mM DTT for 10 min. The eluate was analyzed together with whole-cell lysate and flow-through samples by streptavidin-horseradish peroxidase (HRP) (1:3,000, Invitrogen) by western blot.

### 2.12 Mass spectrometry-based proteomics

Streptavidin magnetic beads containing bead-bound proteins were washed and stored in 100 µL 100 mM triethylammoniumbicarbonat buffer (TEAB). For that, proteins were digested on-bead using trypsin. Proteins were reduced for 1 hour at 55°C using a final concentration of 5 mM Tris(2-carboxyethyl) phosphin (TCEP) followed by reduction for 30 min at room temperature using a final concentration of 10 mM methyl methanethiosulfonate (MMTS). Trypsin was added in a ratio of 1 µg trypsin to 30 µg protein, and proteins were digested overnight at 37°C while shaking. Beads were spun down, and supernatants were transferred to LowBind Eppendorf tubes. Beads were resuspended in 50 µL 250 mM TEAB, spun down, and supernatants were combined and vacuum dried. Pellets were suspended in 50 µL 250 mM TEAB containing 5 mM TCEP and 10 mM MMTS and sonicated for 10 min in an ultrasound water bath. For peptide clean-up, an adapted SP3 protocol was applied (45). For that, 20 µL carboxylate beads were added, and peptides were allowed to bind overnight upon acetonitrile (ACN) addition to a final concentration of at least 95%. Beads were spun down, supernatants were transferred to new LowBind Eppendorf tubes, and 20 µL carboxylate beads were added to increase peptide recovery. Then, beads were combined and washed twice with 100% ACN. Peptides were eluted by using first 20 µL 2% DMSO, followed by a second step with 20 µL Millipore H_2_O. Purified peptides were vacuum dried, and pellets were suspended in 40 µL 0.1% formic acid (FA) and transferred to HPLC vials. For LC-MS/MS analyses, all samples were measured as triplicates on a Dionex UltiMate 3000 n-RSLC system (Thermo Fisher Scientific), connected to an Orbitrap Fusion™ Tribrid™ mass spectrometer (Thermo Fisher Scientific). 200 ng peptides were loaded onto a C18 precolumn (3 μm RP18 beads, Acclaim, 0.075 × 20 mm), washed for 3 min at a flow rate of 6 μL/min, and separated on a 2 μm Pharmafluidics C18 analytical column at a flow rate of 300 nL/min via a linear 60 min gradient from 97% MS buffer A (0.1% FA, 5% DMSO) to 32% MS buffer B (0.1% FA, 5% DMSO, 80% ACN), followed by a 30 min gradient from 32% MS buffer B to 62% MS buffer B. The LC system was operated with the Thermo Scientific SII software embedded in the Xcalibur software suite (version 4.3.73.11, Thermo Fisher Scientific). The effluent was electro-sprayed by a stainless-steel emitter (Thermo Fisher Scientific). Using the Xcalibur software, the mass spectrometer was controlled and operated in the “top speed” mode, allowing the automatic selection of as many doubly and triply charged peptides as possible in a 3-s time window, and the subsequent fragmentation of these peptides. Peptide fragmentation was carried out using the higher energy collisional dissociation mode, and peptides were measured in the ion trap (HCD/IT). MS/MS raw data files were processed via Proteome Discoverer 2.4 mediated searches against the human UniProtKB/SwissProt protein database (release 2021_11) using Sequest HT as a search machine. The following search parameters were used: enzyme, trypsin; maximum missed cleavages, 2; fixed modification, carbamidomethylation (C); variable modification, oxidation (M); precursor mass tolerance, 7 ppm; fragment mass tolerance, 0.4 Da. The false discovery rate (FDR) was set to 0.01. The mass spectrometry proteomics data have been deposited to the ProteomeXchange Consortium via the PRIDE (46) partner repository with the dataset identifier PXD052930.

### 2.13 Data analysis

To generate a final proteome list, only proteins identified in at least three replicates of one group (one group = 3 biological replicates of one condition) with at least two unique peptides were considered. Protein abundancies were normalized to the highest nSMase2 abundancy (**Supplementary Figure S5B**). Sample 40 (H639A-APEX2 TNFα-treated, 5min) was excluded from further analysis due to the overall low amount of detected protein abundancies. Imputation of missing values was performed using Perseus software (Max Planck Institute of Biochemistry, version 2.0.11) (47). To assess the disparity in protein abundance between the untreated and treated samples, we computed the average Log2 Fold Change (Log2FC) of three biological replicates previously averaged over three technical measurements, resulting in a final Log2FC for a specific protein at a given time point. To evaluate the statistical significance of the Log2FCs, we employed Welch’s t-test. To filter possible contaminants from the list of enriched proteins at 2 min, we added control data from the CRAPome database (version 2.0) (48) from 716 proximity-dependent labeling experiments in *H. sapiens*. In the APEX2 data, only proteins with a lower frequency than 75% (178/716) in the CRAPome were allowed. The identified proteins were analyzed by GO enrichment analysis using the Enrich platform (49, 50).

### 2.14 Statistical analysis

Data were analyzed using GraphPad Prism software (GraphPad Software, Inc., version 10) using one-way ANOVA with *post hoc* Tukey test or two-way ANOVA with *post hoc* Sídák test. Schematic figures were created using biorender.com.

## 3 Results

### 3.1 Generation and phenotypic characterization of cell lines expressing the nSMase2-APEX2 fusion protein

**Figure 1:**
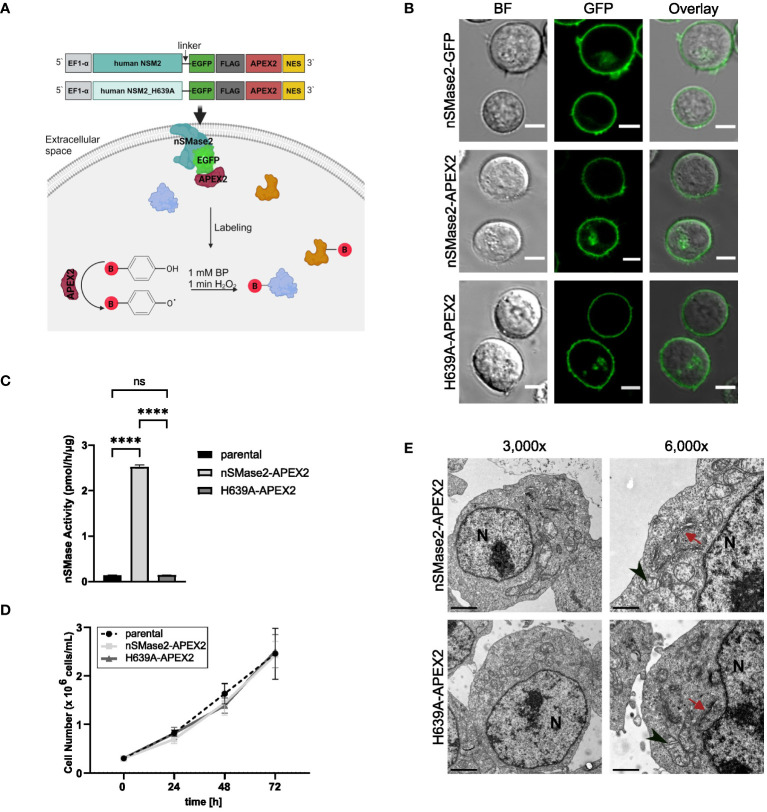
nSMase2 activity and subcellular localization are not affected by C-terminal fusion with APEX2. **(A)** Schematic representation of the designed constructs and APEX2 labeling. **(B)** Subcellular localization of nSMase2 fusion proteins in Jurkat cells. Representative confocal microscopy images of nSMase2-GFP, nSMase2-APEX2 and H639A-APEX2 are shown, scale bars: 5 μm. BF, Brightfield. **(C)** Neutral sphingomyelinase activity measured in cell lysates of parental Jurkat cells or nSMase2-APEX2, H639A-APEX2 expressing cells using BODIPY-FL-C12 Sphingomyelin as a substrate. Mean values with standard deviations of the measurements are shown (n=3), one-way ANOVA with *post hoc* Tukey test was applied for statistical analysis, ****p<0.0001; ns, not significant. **(D)** Growth curves of parental Jurkat, nSMase2-APEX2 and H639A-APEX2 cells. Viable cells were counted after 24, 48, and 72 h of culturing (n=3). **(E)** Representative EM images of nSMase2-APEX2 and H639A-APEX2 cells at low (3,000x) and high (6,000x) magnification, scale bars: 2 μm. Nucleus (N), mitochondria (black arrowheads), and Golgi (red arrows) are indicated.

To monitor rapid activation-dependent alterations in the nSMase2-proximal proteome over time, we employed APEX2-mediated proximity labeling as schematically depicted in **Figure 1A**. Upon its activation by the addition of BP and H_2_O_2_, APEX2 biotinylates proximal proteins that can subsequently be purified from whole cell lysates and identified by MS. To establish Jurkat T cells stably expressing the fusion protein nSMase2-APEX2, we generated a construct wherein human nSMase2, or an enzymatically inactive mutant of nSMase2 (H639A), was C-terminally tagged with APEX2 (**Figure 1A**). This approach was chosen because we aimed to avoid interfering with the correct conformation of nSMase2 at the cytosolic leaflet of the PM, and the N-terminal domain of nSMase2 contains two hydrophobic domains that are crucial for membrane anchoring (7). The enzymatically inactive mutant of nSMase2 should allow to distinguish whether stimulation-induced changes in the nSMase2 proteome correlate with enzymatic activity or result from a scaffolding function. To ensure flexibility and improve the accessibility of nSMase2 within the fusion construct, we included a linker sequence and, to allow monitoring expression and subcellular localization of nSMase2-APEX2 (and its mutant), an EGFP and a Flag-tag, both upstream of the APEX2 coding sequence (**Figure 1A**, upper panels).

When stably expressed in Jurkat cells, nSMase2-APEX2 and H639A-APEX2 mirrored the subcellular localization of nSMase2-tagged with EGFP alone, mainly labeling the PM (**Figure 1B**, **Supplementary Figure S1C**). Moreover, nSMase2-APEX2 and H639A-APEX2 cells did not differ in their expression level verified by measuring EGFP intensities and FLAG tag expression (**Supplementary Figures S1A, B**). As confirmed by *in vitro* assays using fluorescence-labeled sphingomyelin as a substrate, nSMase activity was substantially enhanced in Jurkat cells expressing nSMase2-APEX2 but not in the H639A-APEX2 mutant cells (**Figure 1C**). Furthermore, overexpression of both, enzymatically active and inactive nSMase2 in Jurkat cells did not detectably affect cell proliferation rates (**Figure 1D**) nor did continuously enhanced nSMase2 activity and thereby ceramide release cause abnormalities in the overall architecture of membranous compartments as determined by electron microscopy (EM) (**Figure 1E**, **Supplementary Figures S2A, B**). Taken together, this confirms that the C-terminal APEX2 does not affect subcellular localization and enzymatic activity of nSMase2 and, therefore, is suitable for investigating the nSMase2-proximal protein environment in Jurkat cells.

### 3.2 TNFα stimulation causes nSMase2 activity-dependent membrane ceramide elevation in nSMase2-APEX2 Jurkat cells

**Figure 2:**
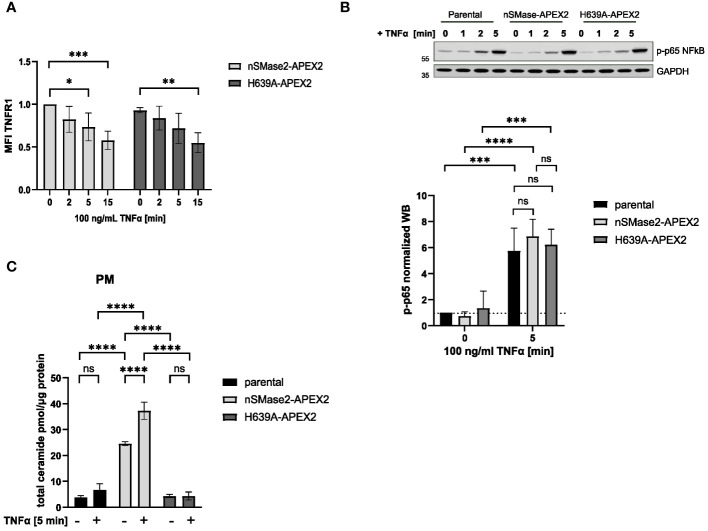
nSMase2-APEX2 fusion protein mediates increase of PM ceramides after TNFα stimulation. **(A)** Surface expression of TNFR1 after stimulation with 100 ng/mL TNFα for 0, 2, 5, 15 min nSMase2-APEX2 and H639-APEX2 cells was assessed by flow cytometry, measuring the mean fluorescence intensity (MFI) using a TNFR1 specific antibody. **(B)** Relative phospho-NFκB p65 levels in nSMase2-APEX2, H639A-APEX2 and parental cell lysates in response to stimulation with 100 ng/mL TNFα for 0, 2 and 5 minutes as assessed by Western blot and normalized to GAPDH levels. **(C)**, Total ceramide levels in lipid extracts of PM fractions from untreated and treated (100 ng/mL TNFα, 5 min) parental, nSMase2-APEX2 or H639A-APEX2 cells as assessed with HPLC-MS/MS. Mean values with standard deviations of the measurements are shown (n=3–4), statistical significance was analyzed by 2-way ANOVA, with *post hoc* Sídák test, ****p<0.0001, ***p<0.001, **p<0.01, *p<0.05, ns, not significant. PM, plasma membrane.

As we aimed to record activation-dependent changes in the nSMase2 proximal proteome, we tested whether nSMase2-APEX2 cells could respond to the established exogenous nSMase2 activator TNFα regarding early signaling events (27, 51). Expression of nSMase2-APEX2 in Jurkat cells did not generally affect early TNFα signaling, as recorded by efficient internalization of TNFR1 and activation of the TNFα downstream effector NFκB that occurred to a comparable extent in nSMase2-APEX2, H639A-APEX2 and parental cells (**Figures 2A, B**). TNFα stimulation activates both PM-localized nSMase2 and endosomal acid sphingomyelinase (ASM). To assess TNFα-induced nSMase2 activation and subsequent PM proximal ceramide production, ceramide levels were determined by HPLC-MS/MS in PM fractions of parental, nSMase2-APEX2, and H639A-APEX2 cells following stimulation. The overexpression of enzymatically active nSMase2 led to elevated ceramide levels, which were further increased by 1.5-fold upon 5 minutes of TNFα stimulation (**Figure 2C**). Similarly, parental cells showed an increase in PM ceramides upon TNFα stimulation (1.8-fold). Still, the change was not significant due to the relatively low activity of endogenous NSM and experimental variations. No increase in ceramides was detected in H639A-APEX2 expressing cells independent of TNFα stimulation, confirming the loss of enzymatic activity of the mutant version of nSMase2. These analyses demonstrate that the nSMase2-APEX2 fusion protein is metabolizing SM and can respond to TNFα stimulation by enhanced ceramide release and thus represent a suitable system to study TNFα activation-mediated nSMase2 proteome alterations.

### 3.3 APEX2-catalyzed labeling and affinity purification of biotinylated proteins in Jurkat cells

**Figure 3:**
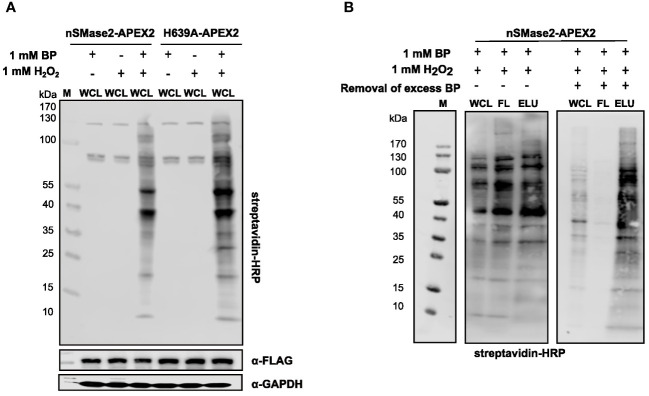
Characterization and optimization of the experimental conditions for APEX2-catalyzed labeling and affinity purification of biotinylated proteins in Jurkat cells. **(A)** Representative analysis of APEX2-, BP-, and H_2_O_2-_dependent generation of biotinylated proteins in the lysates of nSMase2-APEX2 or H639A-APEX2 cells by streptavidin-HRP western blot. α-FLAG antibodies and α-GAPDH antibodies show equal construct expression and protein loading, respectively. **(B)** Optimization of affinity purification of biotinylated proteins in nSMase2-APEX2 and H639A-APEX2 cells. Labeling reaction was initiated, and lysates were either directly incubated with streptavidin magnetic beads (left panel) or filtered using 3 kDa size exclusion columns followed by incubation with streptavidin magnetic beads (right panel). Biotinylated proteins in whole cell lysates (WCL), flow-through (FL), and eluates (ELU) were assessed by streptavidin-HRP western blot. H_2_O_2_, hydrogen-peroxide; BP, biotin phenol; M, marker.

To test the peroxidase activity of APEX2 in the nSMase2 fusion proteins, suspension cells were incubated with BP for 30 min and, for activation of the catalytic biotinylation reaction, treated with 1 mM H_2_O_2_ for 1 min. Treatment with BP and H_2_O_2_ induced extensive protein biotinylation in both nSMase2-APEX2 and H639A-APEX2 Jurkat cells. In contrast, only the endogenous biotinylated proteins (at 130, 75, and 72 kDa) were detected upon omission of BP or H_2_O_2_ (**Figure 3A**). After enrichment and purification of biotinylated proteins using streptavidin beads, biotinylated proteins were detected in the lysate (WCL), however, did not enrich in the eluate (ELU) (**Figure 3B**, left panel). Instead, they were found in the flowthrough fraction (FL), indicating inefficient binding to the streptavidin beads. This may be due to the carry-over of unbound BP exceeding the binding capacity of the streptavidin beads, thereby interfering with the capture of biotinylated proteins. We thus depleted excess BP by filtration of the lysate using ultrafilters with a 3 kDa molecular weight exclusion before the streptavidin affinity purification. Importantly, filtration substantially improved the enrichment of biotinylated proteins in the ELU and resulted in a near-complete removal of biotinylated proteins from the FL (**Figure 3B**, right panel). This strategy was essential for APEX2-dependent proximity labeling in Jurkat cells and may also benefit other cell systems.

### 3.4 Time-resolving proteomic analyses of nSMase2 proximal proteins in response to TNFα stimulation

**Figure 4:**
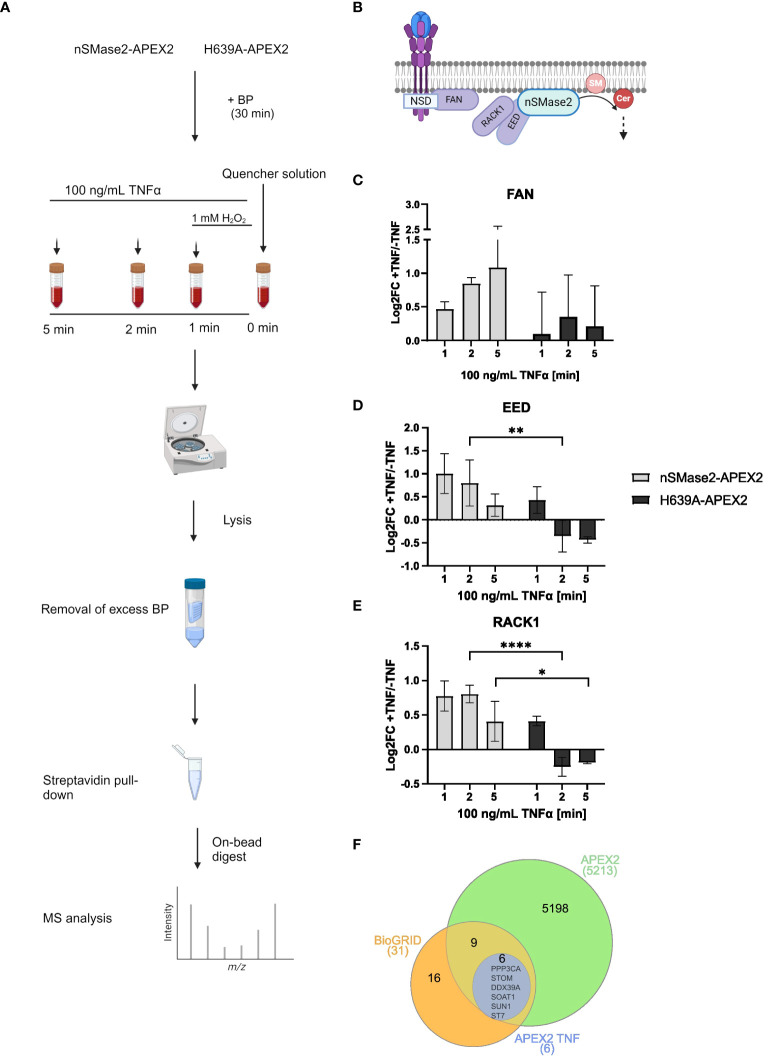
Time resolving proteomic analyses of nSMase2 nanoenvironment in response to TNFα stimulation. **(A)** Experimental workflow for label-free quantitative proteomic analysis of nSMase2 proximal proteins. **(B)** Schematic illustration of known nSMase2-interacting proteins in Jurkat cells in response to TNFα. SM, sphingomyelin; Cer, ceramide. Protein abundance and relative enrichment (Log2Fold Change (FC)) for the known nSMase2-interacting proteins FAN **(C)**, EED **(D)** and RACK1 **(E)** in nSMase2-APEX2 and H639A-APEX2 cells 1, 2 and 5 min after TNFα (100 ng/mL) stimulation, as compared to untreated nSMase2-APEX2 or H639A-APEX2 cells. Mean values with standard deviations from three independent experiments are shown, and statistical significance is indicated (2-way ANOVA, ****p<0.0001, **p<0.01, *p<0.05). **(F)** Venn diagram showing the overlap between nSMase2 interactors reported by BioGRID (orange) and proteins identified in this study using APEX2 labeling (green). Proteins that show increased proximity upon TNFα stimulation are shown in blue.

To study the proximal proteome of nSMase2 and its temporal changes upon early TNFα activation, cell lysates from nSMase2-APEX2 and H639A-APEX2 cultures after 1, 2, and 5 min of TNFα stimulation were prepared and analyzed using unbiased high-resolution liquid chromatography-coupled mass spectrometry (LC-MS) (**Figure 4A**). Purified biotinylated proteins were identified and quantified using a label-free proteomic approach, and data was obtained from three independent biological replicates. Our analyses resulted in the identification of ~5,500 cellular proteins in all replicates (**Supplementary Figure S3**). A cluster map analysis using raw MS data of these proteins confirmed the impact of both nSMase2 activity and TNFα treatment on measured protein abundances (**Supplementary Figure S4**). After identification, filtering (**Supplementary Figure S5A**), and normalization to nSMase2 abundance (**Supplementary Figure S5B**), we calculated the enrichment of proteins in the TNFα stimulated cells versus that of unstimulated controls for each condition (**Supplementary Table S1**).

First, we screened for nSMase2 interactors known to be recruited in response to TNFα in Jurkat cells (**Figure 4B**) (27). The adapter protein FAN was significantly 1.42-fold enriched (Log2FC=0.47; p=0.0006) in nSMase2-APEX2 cells stimulated with TNFα compared to untreated cells at 1 min, and enrichment increased time-dependently confirming that FAN localizes nSMase2-proximal upon TNFα stimulation (**Figure 4C**, **Supplementary Table S1**). The polycomb protein EED was 2-fold enriched (Log2FC=1.00; p=0.07) near nSMase2-APEX2 at 1 min of stimulation compared to untreated cells (**Figure 4D**, **Supplementary Table S1**). EED is known to recruit RACK1 to nSMase2, and, accordingly, our data also revealed TNFα-induced 1.76-fold enrichment of RACK1 peaking at 2 min (Log2FC=0.81, p=0.02) (**Figure 4E**, **Supplementary Table S1**) (27). Our data thus confirm the formation of the nSMase2-FAN-EED-RACK1 protein complex within the first 5 minutes of TNFα stimulation (**Figures 4B–E**) (27). Interestingly, enrichment of EED and RACK1 was significantly less pronounced in the absence of nSMase2 activity in H639A-APEX2 compared to nSMase2-APEX2 cells. Here, enrichment of both EED and RACK1, while detectable at 1 min of TNFα stimulation, was already lost after 2 min of stimulation (**Figures 4D, E**, **Supplementary Table S1**), indicating that complex association depends on nSMase2 activity. In contrast, we did not observe a drastic loss of FAN at 2 and 5 min in the enzymatically inactive H639A-APEX2 cells. Instead, FAN remained enriched at those time points, although this trend was not statistically significant (**Figure 4C**, **Supplementary Table S1**). This indicates that the association of FAN with the complex may be at least partially independent of nSMase2 activity, which is consistent with the reported role of FAN to act upstream of the EED/RACK1/nSMase2 complex. TNF-R1 was not detected in the MS analysis, which may reflect an overall low recovery of complex membrane-resident proteins in conventional MS or a compartmentalization of TNF-R1 and the active nSMase2 signalosome at a distance beyond that covered by APEX2 labeling.

The BioGRID database reports 30 interactors for nSMase2 that were primarily identified through affinity capture-MS studies (52, 53). Indeed, our proximity screening could identify 15 of these proteins supporting the interactions (**Figure 4F**). Notably, most of these reported interactions were independent of TNFα signaling, as their enrichment did not change upon TNFα stimulation. Still, six known interactors of nSMase2 (PPP3CA, STOM, DDX39A, SOAT1, SUN1, ST7) showed significant TNFα-induced enrichment (Log2FC>1, p<0.05) demonstrating that TNFα enhances nSMase2-proximity for these proteins (**Figure 4F**). Amongst those, PPP3CA, the regulatory subunit of PP2B (Ca^2+^/calmodulin-dependent serine/threonine phosphatase calcineurin) was significantly enriched in the proximity of enzymatically active nSMase2 at all time points (**Supplementary Figure S6**, **Supplementary Table S1**). The result indicates that TNFα signaling fosters an early and activity-dependent interaction of nSMase2 and calcineurin in Jurkat cells, corroborating previous observations that described an interaction of nSMase2 and calcineurin in bronchial epithelial (HBE1) cells (54). In summary, protein identification and quantification by MS, along with bioinformatic analysis, revealed reported nSMase2 interactors and significant TNFα-induced enrichment of FAN, EED, and RACK1, thus validating the reliability and effectiveness of our APEX2-based approach.

### 3.5 TNFα induces highly dynamic enzymatic activity-dependent alterations in the nSMase2-proximal protein network

**Figure 5:**
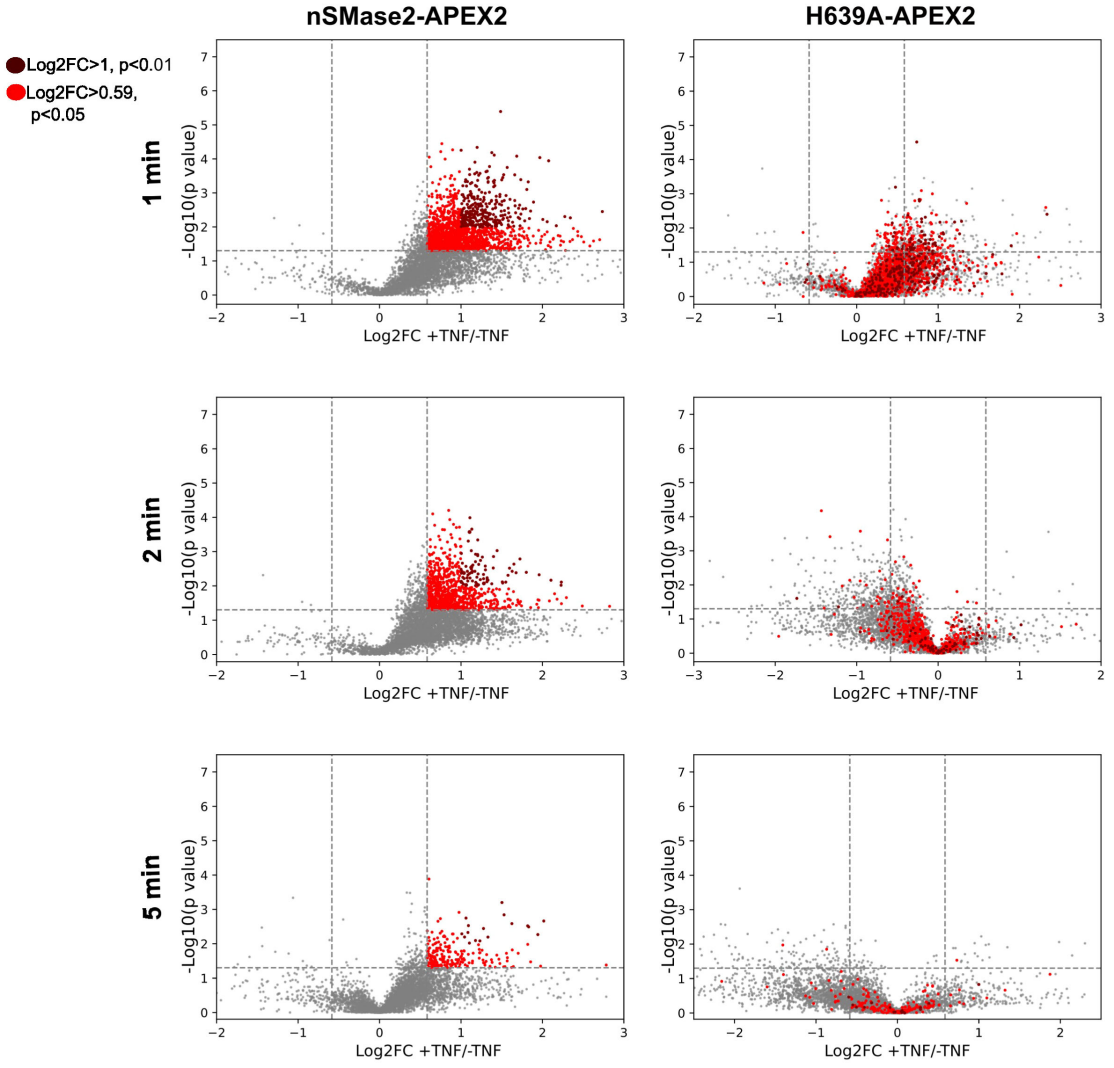
TNFα induces highly dynamic, enzymatic activity-dependent changes in the nSMase2-proximal protein network. Volcano plots showing the enrichment (Log2FC) of identified and quantified proteins in nSMase2-APEX2 cells (left) and H639A-APEX2 cells (right), stimulated with 100 ng/mL TNFα for 1, 2, and 5 min. Protein abundance is normalized to nSMase2 abundance (see **Supplementary Figure S5**). Fold changes (Log2FC) of nSMase2-APEX2 + TNFα/nSMase2-APEX2 -TNFα and H639A-APEX2 + TNFα/H639A-APEX2 -TNFα for each time point are plotted against the p-values (-log10, Welch`s t-test). The data from three (n=3) independent biological replicates are shown, with each sample measured in three technical replicates. Proteins in nSMase2-APEX2 cells with Log2FC>0.05 and -log10 (p value) > 1.3 or Log2FC > 1 and -log10 (p-value) > 2 after TNFα treatment are highlighted in light red and dark red, respectively, in the nSMase2-APEX2 and H639A-APEX2 plots.

Next, all identified proteins in the different conditions were visualized as volcano plots, plotting the FC (log2) of TNF-treated *versus* non-treated samples against the p-values (-log10) from three independent biological replicates. TNFα treatment resulted in major dynamic changes in the nSMase2-APEX2-proximal proteome with robust and reproducible enrichment of proteins, particularly profound after 1 and 2 minutes after treatment (**Figure 5**, left panels). The presence of hundreds of proteins enriched in the proximity of nSMase2-APEX2 following TNFα stimulation indicates a rapid remodeling of protein networks at the PM upon TNFα stimulation. The apparent decrease of nSMase2 proximal proteins seen 5 min after TNFα treatment most likely indicates termination of the first wave of signaling initiation involving PM resident nSMase2 (**Figure 5**, left panels). To assess the effect of nSMase2 enzymatic activity on its proximitome, the proteins enriched in the nSMase2-APEX2 cells were compared to the proteins within the microenvironment of H639A-APEX2 cells for each stimulation timepoint (**Figure 5**, right panels). We defined a cut-off to reduce background protein interactions and to evaluate only candidates that strongly integrate into the nSMase2 proximal protein network. We selected proteins that were more than 1.5-fold enriched (log2FC >= 0.59) compared to TNFα untreated conditions and a p-value below 0.05 (-log10(p) >= 1.3) and highlighted those in red (**Figure 5**). Within these criteria, we found known TNFα induced interactors of nSMase2 like RACK1, as well as proteins associated with TNFα-activated MAPK and NFκB signaling cascades (**Supplementary Table S1**).

Interestingly, stimulation-dependent proximity enrichment of the proteins observed in nSMase2-APEX2 (highlighted in red) appeared much less pronounced in H639A-APEX2 cells and even seemed to be de-enriched at 2 and 5 minutes, suggesting a dependency on nSMase2enzymatic activity. Moreover, similar to our observations for EED and RACK1, most of the proteins initially detected within the proximity of catalytically inactive H639A-APEX2 in TNFα stimulated cells were lost after 2 and 5 minutes of stimulation (**Figure 5**, right panels).

Together, these results confirm that our strategy allows robust resolution of dynamic changes in the nSMase2 protein environment within the first 5 minutes of TNFα stimulation. Our observations suggest that nSMase2 activity may be particularly important in sustaining proximity with proteins after stimulation (**Figure 5**, 2, and 5 min), while this does not fully apply to their initial recruitment (**Figure 5**, 1 min).

### 3.6 The phosphorylation of MAPK p38 and JNK/SAPK is not affected by proximity to nSMase2-APEX2

**Figure 6:**
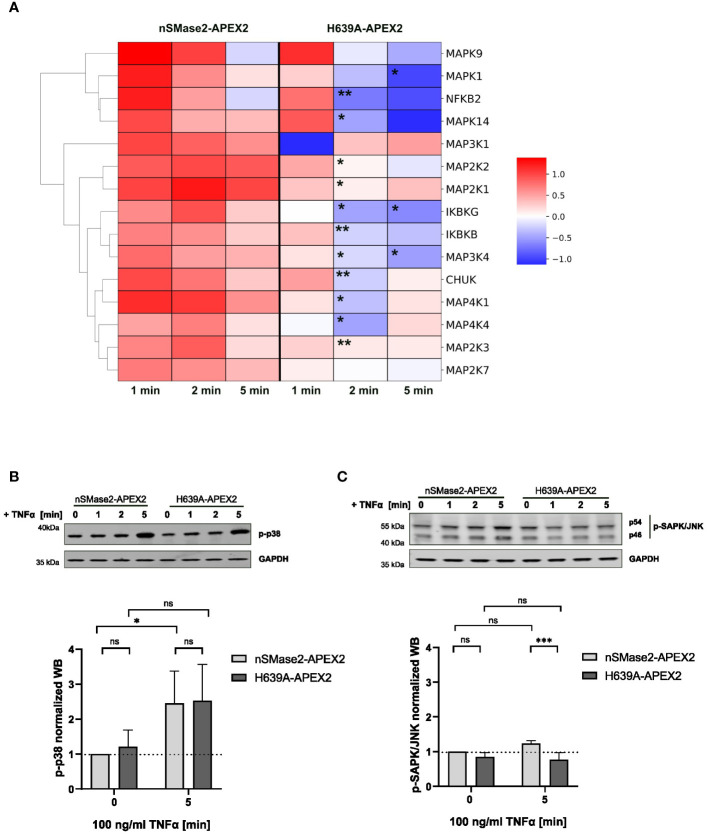
MAPK and NFκB signaling components are enriched in the proximity of nSMase2-APEX2 in TNFα-stimulated cells. **(A)** Clustermap of proteins associated with MAPK or NFκB signaling that are significantly enriched at nSMase2 (Log2FC>0.59, p<0.05) in at least one time point of TNFα stimulation in nSMase2-APEX2 cells. The Log2FC values are indicated by the colored scale, where dark red indicates a value>1 and blue indicates a value < -1. Stars indicate significant differences between protein enrichment in nSMase2-APEX2 and H639A-APEX2 cells; Welch`s t-test, n=3, *p<0.05, **p<0.01. Western blot phosphorylation analysis of **(B)** p38 and **(C)** JNK/SAPK in nSMase2-APEX2 and H639A-APEX2 cell lysates in response to stimulation with 100 ng/mL TNFα for 0, 1, 2, and 5 minutes. Phosphorylation signals were normalized to GAPDH levels. Mean values with standard deviations for three independent experiments (n=3) are shown. Statistical analysis by 2-way ANOVA, with *post hoc* Sídák test: ***p<0.001, **p<0.01, *p<0.05, ns, not significant.

MAP kinase and NFkB signaling pathways are activated early after TNFα binding to the cellular receptors, contributing to its inflammatory activity (55, 56). Our labeling data provided the opportunity to analyze the proximity of components of the MAPK and NFkB pathways to nSMase2 (**Supplementary Table S1**). We found 15 MAPK or NFκB associated proteins that were significantly enriched in at least one time point of stimulation. Next, we evaluated their enrichments within the nSMase2 proximal proteome over time and whether these depended on nSMase2 enzymatic activity (**Figure 6A**). Ablation of nSMase2 enzymatic activity led to significant reduction of the TNFα-induced enrichment at 2 and 5 minutes after TNFα application (**Figure 6A**). This was consistent with our observation made for RACK1 and EED that sustained proximity requires nSMase2 activity (see **Figures 4D, E**).

We found an enrichment of known members of the three MAPK signaling pathways: p38 (MAP2K3, MAPK14), JNK (MAP4K4, MAP4K1, MAP2K7, MAPK9) and ERK (MAP2K2, MAP2K1, MAPK1), as well as MAPKs with broader functions in more than one cascade (MAP3K4, MAP3K1) (**Figure 6A**). MAPK14 (p38α-MAPK) was shown to be upstream of nSMase2 in TNFα stimulated A549 epithelial cells (20). Other published data showed that nSMase2 and ceramide production were involved in TNFα mediated inflammatory responses in monocytes and macrophages. The authors showed impaired JNK and p38 MAPK phosphorylation in cells lacking nSMase2 expression (22). However, the proximity of MAPKs to nSMase2-APEX2 does not reveal whether nSMase2 has a regulatory role in TNFα-mediated activation of MAPKs in Jurkat cells. To answer that, we analyzed TNFα-induced phosphorylation of p38 MAPK (Thr180/Tyr182) and JNK/SAPK (Thr183/Tyr185) over time, focusing on the 5-minute stimulation period when the effects were most pronounced (**Figures 6B, C**). TNFα-induced phosphorylation levels of p38 did not differ in nSMase2-APEX2 and H639-APEX2 cells, indicating that p38 phosphorylation was not affected by the proximity to nSMase2 or nSMase2-APEX2-dependent ceramide production in TNFα stimulated Jurkat cells (**Figure 6B**). Unlike p38 MAPK, we observed only a minor increase in the phosphorylation of SAPK/JNK, another member of the MAPK family, after TNFα stimulation of nSMase2-APEX expressing cells indicating a limited, if any, effect of nSMase2-APEX expression and proximity on SAPK/JNK activation (**Figure 6C**).

For the NFκB pathway, TNFα-induced enrichment was observed for members of the IKK complex (IKBKG (NEMO), IKBKB, CHUK) and the transcription factor NFKB2/p52 (**Figure 6A**). Like MAPK, the observed proximity of members of the NFκB signaling pathway with nSMase2 did depend on nSMase2 enzymatic activity. However, this did not translate into early nSMase2 activity-related effects on NFkB signaling as the phosphorylation of RELA/p65 in enzymatically inactive H639A-APEX2 cells corresponded to that of nSMase2-APEX2 cells (see **Figure 2B**).

The proximity analysis revealed an early TNFα and nSMase2 activity-dependent increase in the proteins associated with MAPK and NFκB signaling pathways in the vicinity of nSMase2-APEX2. However, this did not affect the overall p38 and NFκB/p65 activation levels.

### 3.7 TNFα induces enrichment of proteins involved in vesicle-mediated transport in the nanoenvironment of nSMase2

**Figure 7:**
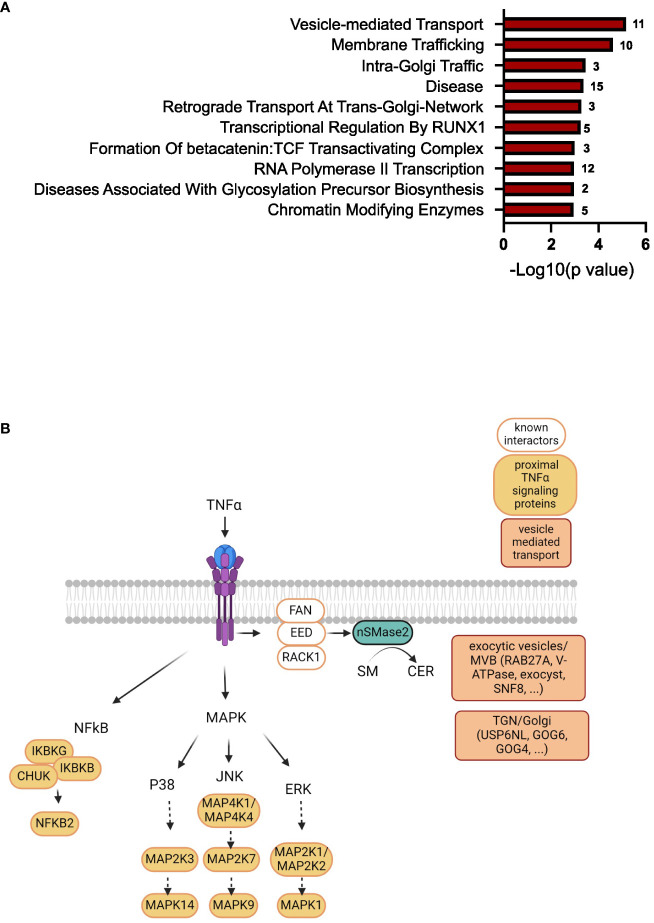
TNFα induces enrichment of proteins involved in vesicle-mediated transport in the nSMase2 nanoenvironment. **(A)** Gene ontology analysis using ENRICHR software (Reactome pathway) of significantly enriched (Log2FC>1, p<0.01) proteins at 2 minutes after TNFα stimulation in nSMase2-APEX2 cells. The number of proteins found in our dataset that are associated with these processes is shown to the right of each bar. **(B)** Scheme of the nSMase2 protein nanoenvironment in TNFα-stimulated Jurkat cells revealed by proximity labeling. nSMase2 enzymatic activity is required for the detection of the proteins shown in the proximity of nSMase2-APEX2. Published interacting proteins are depicted in white, identified proximal TNFα signaling proteins of MAPK and NFκB pathways in orange, and proteins associated with vesicle-mediated transport are highlighted in red.

To further narrow down the list of potential nSMase2 proximal proteins, a more stringent cut-off was applied, targeting highly enriched proteins in nSMase2-APEX2 cells with a least a 2-fold (log2FC >= 1) enrichment and a p-value of p<0.01 (-log10(p) >= 2) as highlighted in dark red (**Figure 5**). In total, the stringent cut-off revealed 310 proteins at 1 min, 82 proteins at 2 min, and 17 proteins at 5 min after TNFα stimulation to be significantly enriched (**Supplementary Table S2**). To further evaluate proteins and processes dependent on nSMase2 activity, we shortlisted the 2 min data set using the more stringent cut-off (2-fold enrichment, p<0.01) and by eliminating potential background proteins using the CRAPome database (48). The remaining 66 identified proteins could be grouped into Reactome pathways according to the ENRICHR gene ontology software, with components of vesicle-mediated transport (11 of 66 proteins, 17%) and membrane trafficking (10 of 66 proteins, 15%) as the most highly enriched (**Figure 7A**, **Supplementary Table S2**). We further categorized the proteins associated with vesicle-mediated transport based on their compartment-specific localization according to literature and annotations in the STRING database (57) (**Table 1**).

**Table 1:** Compartment-specific localization of proteins associated with vesicle-mediated transport.

Protein name	Log2FC	p-value	compartment
**USP6NL**	1.00	0.003	Early/recycling endosomes (58, 59), trans-Golgi network, Golgi membrane, cytoplasmic vesicle
**COG6**	1.00	0.008	Trans-Golgi network (60), Golgi membrane
**CUX1**	1.18	0.01	Golgi membrane, COPI vesicle (61)
**COG4**	1.24	0.007	Trans-Golgi network, Golgi membrane
**USO1**	1.08	0.004	Recycles between cytosol and Golgi, transport vesicle, Golgi membrane, cytoplasmic vesicle
**RAB27A**	1.28	0.008	Exocytic vesicles (62), transport vesicle,
**HYOU1**	1.55	0.008	ER to Golgi, cytoplasmic vesicle
**SYNJ2**	1.13	0.004	Transport vesicle, cytoplasmic vesicle, endocytic vesicles (63)
**SNF8**	1.04	0.004	Endosome membrane, ESCRT-II component, cytoplasmic vesicle, MVB
**EXOC2**	1.80	0.004	vesicle tethering complex (exocyst complex),
**TBC1D15**	1.01	0.008	Endosome (64)

Among these vesicular proteins, we identified key components of the multivesicular body (MVB) secretory machinery, including Ras-related protein Rab-27A (RAB27A), vacuolar-sorting protein SNF8 (SNF8), and exocyst complex component 2 (EXOC2). Interestingly, we found several subunits of the vacuolar ATP-dependent proton pump (V-ATPase) that were significantly enriched in the 1 and 2 min nSMase2-APEX2 dataset: ATP6V0D1, a subunit from the transmembrane domain, and ATP6V1E1, ATP6V1F, ATP6V1H subunits from the cytosolic domain (Tab S1, highlighted in yellow). These subunits belong to the V-ATPase complex, a crucial regulator of endosomal acidification and exosome secretion. In HeLa cells, its activity within the MVB compartment is counteracted by nSMase2 in response to TNFα, leading to increased exosome secretion (23). Our data imply that nSMase2-mediated suppression of endosomal acidification is possibly initiated within the first 2 minutes after TNFR engagement. Importantly, we did not detect significant enrichment of V-ATPase subunits in the protein environment of H639A-APEX2, indicating that sphingomyelinase activity is crucial to stabilize the nSMase2 proximity to the proteins of the endosomal compartment. Noteworthy, UNC13D, which regulates cytotoxic granule secretion in lymphocytes, and additional exocyst complex members (EXOC3, EXOC4, EXOC6, EXOC8) were identified to be significantly enriched at 1 min (**Supplementary Table S1**, highlighted in yellow). Our detected proximity between nSMase2 and proteins involved in the MVB secretory machinery aligns with previous research on the role of nSMase2 in regulating TNFα-induced MVB-derived exosomes (23). Furthermore, we find proteins associated with vesicular transport at the Golgi or trans-Golgi network, including USP6 N-terminal-like protein (USP6NL), conserved oligomeric Golgi complex subunit 6 (COG6), conserved oligomeric Golgi complex subunit 4 (COG4), general vesicular transport factor p115 (USO1). This most likely reflects the dynamic stimulation-induced communication of MVBs with other organelles or compartments, including the Golgi and trans-Golgi network. Taken together, beyond the well-known nSMase2 interactors and TNFα signaling proteins of the NFκB and MAPK pathways, TNFα signaling significantly enriches proteins associated with vesicular transport in the nanoenvironment of nSMase2 and this relies on nSMase2 activity (**Figure 7B**). Among those, we detected proteins that are related to vesicle-mediated transport originating from different compartments like Golgi, trans-Golgi network, and MVB/exocytic vesicles.

## 4 Discussion

Metabolic breakdown of sphingomyelin by nSMase2 and subsequent ceramide production was shown to be an essential part in regulating TNFR signaling (22, 24–27, 65). To shed the light on dynamic alterations of the nSMase2 proximal proteome early after TNFα exposure, we chose the APEX2-based proximity labeling technique coupled with affinity purification and quantitative MS. When stably expressed in Jurkat cells, the recombinant nSMase2-APEX2 protein maintained its enzymatic activity, which, as anticipated, exceeded that of the unmodified parental cells in whole cell extracts (**Figures 1**, **2**).

Technically, we followed the established protocol for using the APEX2 system for proteome analysis, which demanded, however, modifications allowing for efficient recovery of biotinylated proteins from cell lysates. Removing excess BP from the lysate substantially improved the recovery of biotinylated proteins, and we recommend this to be included in proximity labeling protocols (**Figure 3**).

Protein identification, quantification by MS, and bioinformatic processing revealed a significant TNFα-induced enrichment of known nSMase2 interacting proteins, thus validating the robustness of our APEX2-based approach (**Figure 4**) (26, 27). Earlier studies identified the NSD in the cytosolic tail of TNFR1, which was later found to recruit FAN directly (24). FAN, in turn, is essential to couple and activate nSMase2 by further recruiting RACK1 and EED (25–27). EED directly interacts with nSMase2 via its C-terminus, thereby orchestrating the assembly of nSMase2 into the multi-protein complex with TNFR1 and FAN. In line with FAN, EED, and RACK1 being crucial for early activation of nSMase2 (27), we found these proteins enriched in the proximity of nSMase2 already at 1 min after TNFα stimulation. The early formation of this complex was only partially dependent on nSMase2 activity. In contrast, EED and RACK1 proximity to nSMase2 was lost after 2 minutes of stimulation of cells expressing an enzymatically inactive version ofnSMase2: H639A (**Figures 4D, E**). The lack of identification of the TNFR itself may reflect limited solubility and suboptimal retrieval of transmembrane proteins in conventional MS (66).

In addition, in our proximity data, we could identify 50% (i.e., 15 proteins) of the nSMase2 interactors reported by BioGRID, with six out of 15 showing a significant enrichment upon TNFα induction (**Figure 4F**). Among them was PPP3CA, the catalytic subunit of the phosphatase calcineurin. In HB1 cells, calcineurin was previously found to interact directly with nSMase2 under steady-state conditions (54). Moreover, under oxidative stress, calcineurin was shown to dissociate from nSMase2, thereby regulating the phosphorylation status of nSMase2 and thus modulating its activity. Reduced calcineurin/nSMase2 interaction, thereby, activation of nSMase2 under oxidative stress was monitored after 30 min, and thus late after treatment (54). Here, we show that calcineurin is most likely interacting with nSMase2 in Jurkat cells, too, and, surprisingly, this interaction was enhanced already early after TNFα treatment. It is quite possible that the regulation of nSMase2 phosphorylation early after its activation is differentially regulated. The fact that the nSMase2/calcineurin interaction depends on nSMase2 enzymatic activity strongly suggests that, unlike for oxidative stress conditions, calcineurin most likely functions downstream of nSMase2 in response to TNFα. Furthermore, differences in interaction and phosphorylation dynamics of the nSMase2/calcineurin complex may depend on the stimulation strategy and the cellular background. Notably, the role of nSMase2 phosphorylation regulating its activity by cellular proteins was supported by the finding that the enzymatic activity of a truncated recombinant nSMase2 protein lacking the intervening domain, and thereby, all phosphorylation sites (which was used for crystallization) was identical to that of the full-length nSMase2 in an *in vitro* assay (1).

The activation of nSMase2 in response to oxidative stress may raise concerns about the potential impact of H_2_O_2_, used as a substrate for the APEX2 reaction, on nSMase2 activity and its proximitome. However, nSMase2 activation in response to H_2_O_2_ has been observed after 30 minutes of treatment, a significantly longer duration than the 1-minute treatment for APEX2 labeling (54). Therefore, we believe that the observed effects are more likely attributed to the activation by TNFα, which we have confirmed to occur within the first 5 minutes, rather than activation by H_2_O_2_.

Our analysis revealed a striking enrichment in nSMase2-proximal proteins in response to TNFα (**Figure 5A**). This unveiled the dynamic nature of TNFα-induced nSMase2 interactions with cellular proteins, with the majority of changes occurring in the first 2 minutes after activation. Remarkably, ablation of the catalytic activity of nSMase2 completely abrogated protein enrichment at 2 and 5 min after TNFα induction, indicating that its activity directly governs the substantial changes in the nSMase2 proximal protein network. With its potential to modify the cytosolic leaflet of the PM membrane, the nSMase2 bears the potential to organize microdomains and, thereby, the formation of receptor-proximal signaling clusters (67). The inability of the enzymatically inactive mutant to maintain proximity could indicate that the stability of TNFα-induced protein complexes at the PM depends on nSMase2 activity, while this does apply only partially to the initial recruitment of these proteins (**Figure 5**). These observations suggest that nSMase2 activity may be essential in the sustainment of TNFR signaling after stimulation. In line with this, nSMase2 activation has been revealed as crucial for the sustainment and propagation of T cell receptor (TCR) signaling, especially regarding microtubular integrity and, thereby, vesicle transport (68). Thus, processes such as initial TCR microcluster formation, CD3ζ phosphorylation, and Lck activation were found unaffected on genetic ablation of nSMase2, while CD3ζ and ZAP-70 phosphorylation could not be sustained, the microtubular system not polarized and stabilized and hence, signal amplification was strongly attenuated.

Ceramide plays a central role in sphingolipid metabolism, and its level is tightly controlled as it is readily converted into ceramide 1-phosphate, sphingomyelin, and more or less complex glycosphingolipid species (69). In the catabolic pathway, ceramide is converted to sphingosine, the precursor to sphingosine 1-phosphate (S1P), which has been implicated in regulating TNFα signaling (70, 71). Since cellular effects downstream of TNFR occur rapidly and – as addressed by us – involve nSMase2 activity, it is most likely that ceramides, rather than their downstream metabolites, are the actual effectors. Accumulation of ceramides has been shown to impact the biophysical properties of cellular membranes substantially, also reflected by the formation of ceramide-enriched platforms at the PM (72–74). These platforms act to segregate receptors, which can be actively included or excluded from these platforms and facilitate recruitment of signaling complexes and thereby signal initiation and propagation.

Though the role of ceramide in regulating NFκB and MAPK signaling cascades downstream of TNFα has been established (22, 75–77), the contribution of nSMase2 is still inconclusive and may depend on cellular backgrounds (51). We rationalized that the nSMase2 protein environment is regulated very early after the initiation of TNFR signaling. Therefore, we aimed to perform the analysis within the first 5 minutes after TNFR stimulation. The study in monocytes and macrophages describes the positive role of TNFR-dependent nSMase2 activation and subsequent ceramide production in sustained TNFR signaling analyzed after 15 minutes of stimulation (22). However, we did not observe any nSMase2 activity-dependent regulation of TNFR-induced phosphorylation of p65 subunit of NFκB (**Figure 2B**) within the first minutes of stimulation, suggesting that signaling initiation is not regulated by the nSMase2-generated ceramides.

Our proximity labeling data set allowed the identification of membrane-proximal TNFα signaling components after stimulation, thereby defining regulatory and functional downstream consequences in Jurkat cells. We identified 15 proteins associated with MAPK and NFκB, of which many are known to be modulated by TNFα (55, 78–80). This implicates a physical closeness for nSMase2 to the proteins of those signaling cascades, thereby strongly supporting previous studies on the role of nSMase2 in these signaling pathways (22, 76, 81). Moreover, our data indicated that nSMase2 activity is required for sustained protein proximity (**Figure 6A**). However, the initial recruitment of p38α (MAPK14) into proximity with nSMase2 and its phosphorylation occurred independently of nSMase2 activity (**Figures 6A, B**). In A549 cells, p38 was identified as an upstream regulator of nSMase2 as the endogenous activity of nSMase2 in response to TNFα stimulation was abolished upon inhibition of p38 MAPK (20). In contrast to published data showing a crucial role of nSMase2 in TNFα-dependent MAPK activation in macrophages (22), our data suggest that p38 phosphorylation is independent of proximity to enzymatically active nSMase2 in Jurkat cells. Still, we cannot exclude MAPK14 as a possible upstream regulator of nSMase2 also in Jurkat cells. Inhibition of nSMase2 prevented TNFα-induced RELA/p65 phosphorylation and NFκB/AP-1 activity in macrophages (22). We did not observe an nSMase2 activity-dependent effect on RELA/p65 phosphorylation in nSMase2-APEX2 or H639A-APEX2 cells during early TNFα stimulation (**Figure 2B**). Nevertheless, we identified several members of the NFκB pathway to be enriched in an nSMase2 activity-dependent manner (**Figure 6A**). Further investigations are needed to understand if the recruitment of IKK complex constituents: IKBKB, IKGKG, and CHUK, or the NFκB subunit NFKB2/p100 in the proximity of nSMase2 has functional consequences at the later stages of TNFα stimulation or reflects the dynamic changes of nSMase2 proximal proteome.

We applied a multi-layered filtering approach to identify nSMase2 proximal partners with high confidence. At 2 minutes after TNFα stimulation, the majority of proteins were associated with intracellular vesicle-mediated transport and exocytic vesicles (**Figure 7A**). A fundamental role of nSMase2 in the biogenesis and release of exosomes has been found in a variety of cell types where pharmacological inhibition, knock-down or knock-out of nSMase2 decreased exosome release (82–84). In response to TNFα stimulation, Choezom et al. demonstrated that nSMase2 modulates small extracellular vesicle (sEV) secretion by regulating V-ATPase activity (23). Corroborating on that, we identified V-ATPase subunits and MVB secretory proteins in the nanoenvironment of nSMase2 upon TNFα stimulation. In addition, ceramide generated by nSMase2 has been reported to serve as a fusion point of N-methyl D-aspartate (NMDA) receptor-laden vesicles with the PM in response to TNFα (18).

We also identified other proteins associated with processes not typically linked to PM and annotated as cytosolic or nuclear [for example, transcriptional regulation by RUNX or chromatin-modifying enzymes (**Figure 7A**, **Supplementary Table S2**)]. At first glance, it was striking that nuclear proteins were found to be enriched. Notably, EED (see **Figure 4B**) functions as one of the regulatory subunits of the polycomb repressive complex 2 (PRC2), a post-translational modifier of chromatin and, thereby, gene expression in the nucleus (85). Considering that fast recruitment of EED to the PM does occur, it cannot be excluded that this also applies to proteins not expected to localize there. Indeed, we identified two more PRC2 complex-associated proteins to be highly enriched, indicating that EED translocation to the PM might also involve the regulatory PCR2 subunit RBBP4 and the facultative subunit JARID2. In addition, the unique morphology of Jurkat T cells, characterized by a relatively large nucleus and a small cytoplasm, may increase the likelihood of labeling nuclear proteins compared to other cell lines. TNF-induced changes might involve the formation of membrane contact sites between the endoplasmic reticulum and the PM, bringing the nucleus within the labeling radius of APEX2.

The limitation of this study is the need to validate the dynamic nature of the protein content in the vicinity of nSMase2. An important question, which stays beyond the scope of this manuscript, is the subcellular localization of nSMase2-APEX2 and its proximal proteins at the initial stages of TNFα stimulation. We have previously shown that most of nSMase2 is localized at the PM, and the smaller intracellular pool of nSMase2 does not associate with the ER or organelles of the secretory pathway (39). However, we have observed the association of nSMase2 with newly formed lipid droplets in a fatty acid-rich environment. We now show a clear PM association for nSMase2-APEX with occasional intracellular localization (**Figure 1B**), which did not increase significantly after TNFα stimulation (**Supplementary Figure S7**). Therefore, we hypothesize that most TNFα-induced nSMase2 microenvironments were detected at the PM. However, we cannot exclude the possibility that high-resolution analysis might reveal some nSMase2-containing intracellular compartments induced by TNFα stimulation in Jurkat cells.

In summary, our work underscores the power of APEX2 to visualize rapid changes in protein complex formation in living cells. By monitoring proximal nSMase2 proteins over time, our study provides a comprehensive overview of the highly dynamic protein network in response to TNFα. We confirmed reported interactors and unveiled novel insights into the role of nSMase2 in the early TNFα-mediated response, suggesting its involvement in early initiated vesicle-mediated transport and membrane trafficking. The ability of nSMase2-generated ceramides to regulate sustained protein enrichment in the response of Jurkat cells to TNFα becomes visible, and we anticipate that this phenomenon may extend to other cell types as well. For future analyses, it will be imperative to integrate the impact of locally induced ceramide on cellular processes for accurate interpretation of experimental data associated with the manipulation of sphingolipid metabolism.

## Data availability statement

The datasets presented in this study can be found in online repositories. The names of the repository/repositories and accession number(s) can be found below: PXD052930 (ProteomeXchange).

## Ethics statement

Ethical approval was not required for the studies on humans in accordance with the local legislation and institutional requirements because only commercially available established cell lines were used. Ethical approval was not required for the studies on animals in accordance with the local legislation and institutional requirements because only commercially available established cell lines were used.

## Author contributions

MS: Data curation, Formal analysis, Investigation, Methodology, Software, Visualization, Writing – original draft. RS: Investigation, Visualization, Writing – review & editing. TH: Methodology, Writing – review & editing. DW: Data curation, Formal analysis, Investigation, Writing – review & editing. FS: Methodology, Writing – review & editing. BK: Funding acquisition, Methodology, Writing – review & editing. CS: Funding acquisition, Methodology, Writing – review & editing. MvH: Data curation, Formal analysis, Investigation, Methodology, Software, Writing – review & editing. LJ: Methodology, Writing – review & editing. SS-S: Conceptualization, Funding acquisition, Project administration, Supervision, Writing – original draft. LD: Funding acquisition, Project administration, Resources, Supervision, Writing – original draft. EA: Conceptualization, Funding acquisition, Project administration, Supervision, Writing – original draft.
