# Supplementary material for: Dynamic changes in the proximitome of neutral sphingomyelinase-2 (nSMase2) in TNFα stimulated Jurkat cells
Source: Front Immunol. 2024 Jul 9;15:1435701. doi: 10.3389/fimmu.2024.1435701 (PMC11263205; doi:10.3389/fimmu.2024.1435701)
Supplement: Supplementary file 3 [file DataSheet_1.docx]

Supplementary Material

# Supplementary Tables

Table S1: Normalized Log2FC mass spectrometry values of all identified proteins by APEX2-mediated proximity labeling in nSMase2-APEX2 and H639A-APEX2 cells. Information about protein name, protein description, unique peptides, logarithmized FC from TNFα treated vs. untreated for 1 min, 2 min, and 5 min, and the respective -log10 (p-value), is given. In addition, proteins from nSMase2-APEX2 cells falling into the defined cut-off (FC>0.59, p<0.05) are summarized in sub-tables.

Table S2: Normalized Log2FC mass spectrometry values of 2-fold significantly enriched proteins. Information about protein name, protein description, unique peptides, logarithmized FC from TNFα treated vs. untreated for 1 min, 2 min (before and after filtering according to CRAPome database), and 5 min, and the respective -log10 (p-value) in nSMase2-APEX2 cells, is given.

# Supplementary Figures


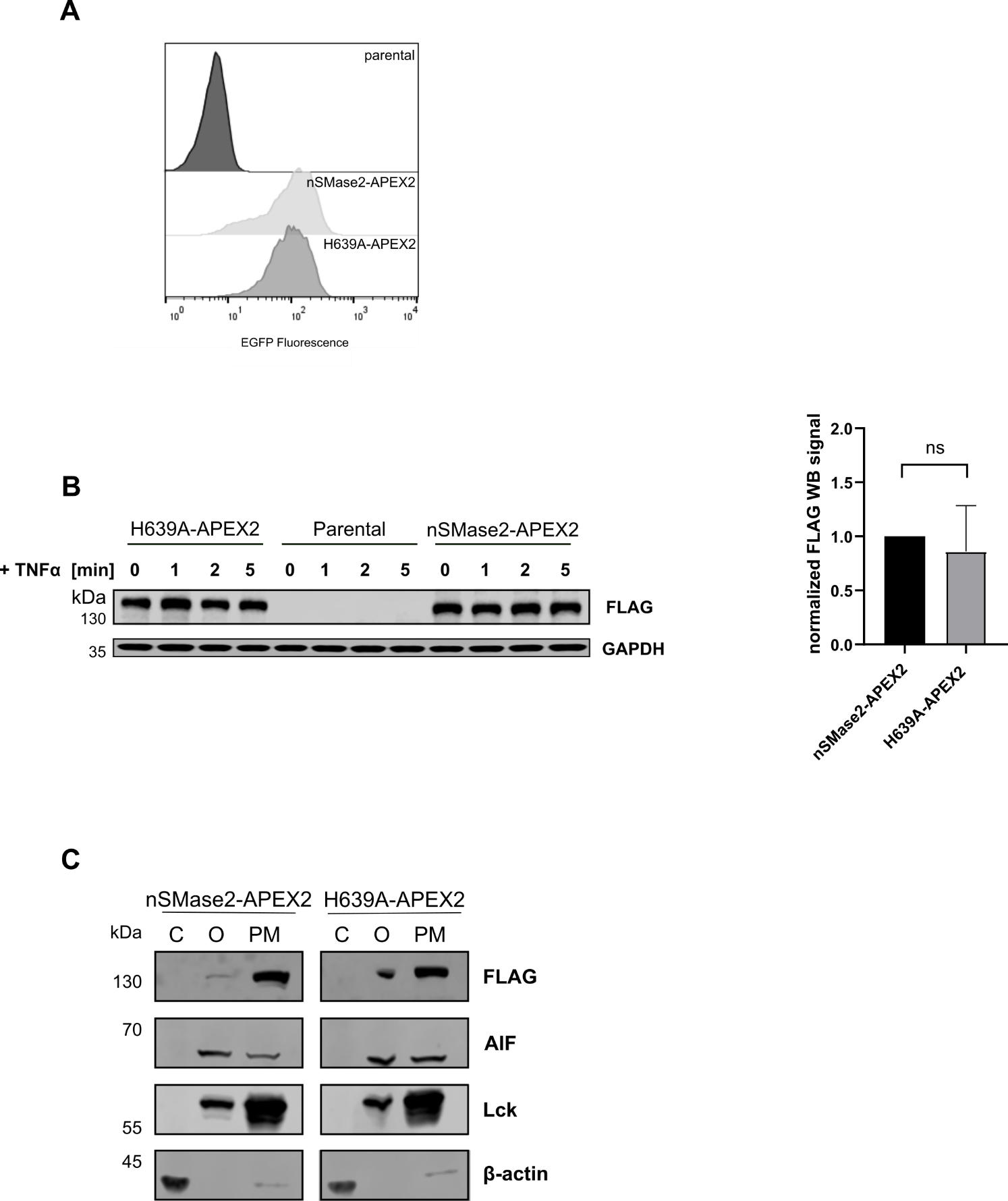


**Supplementary Figure 1.** (A) EGFP expression in nSMase2-APEX2 and H639A-APEX2 cells. EGFP fluorescence intensity in parental(black), nSMase2-APEX2 (light grey), and H639A-APEX2(dark grey) cells as measured by flow cytometry. (B) Western blot of fusion construct expression levels in nSMase-APEX2 and H639A-APEX2 cell lysates using an α-FLAG antibody. Parental cells are shown as a FLAG staining negative control, and cells were stimulated with 100 ng/mL TNFα for the indicated time points. Mean values of three biological replicates from unstimulated cells with standard deviations of the measurements are shown on the right (n=3), unpaired t-test, ns= not significant. (C) Cell fractionation followed by WB analysis. PM is marked by an anti-Lck antibody, O is marked by an anti-AIF antibody, and C is marked by an anti- β -actin antibody). C, Cytoplasmic fraction; O, organelle fraction; PM, plasma membrane fraction.


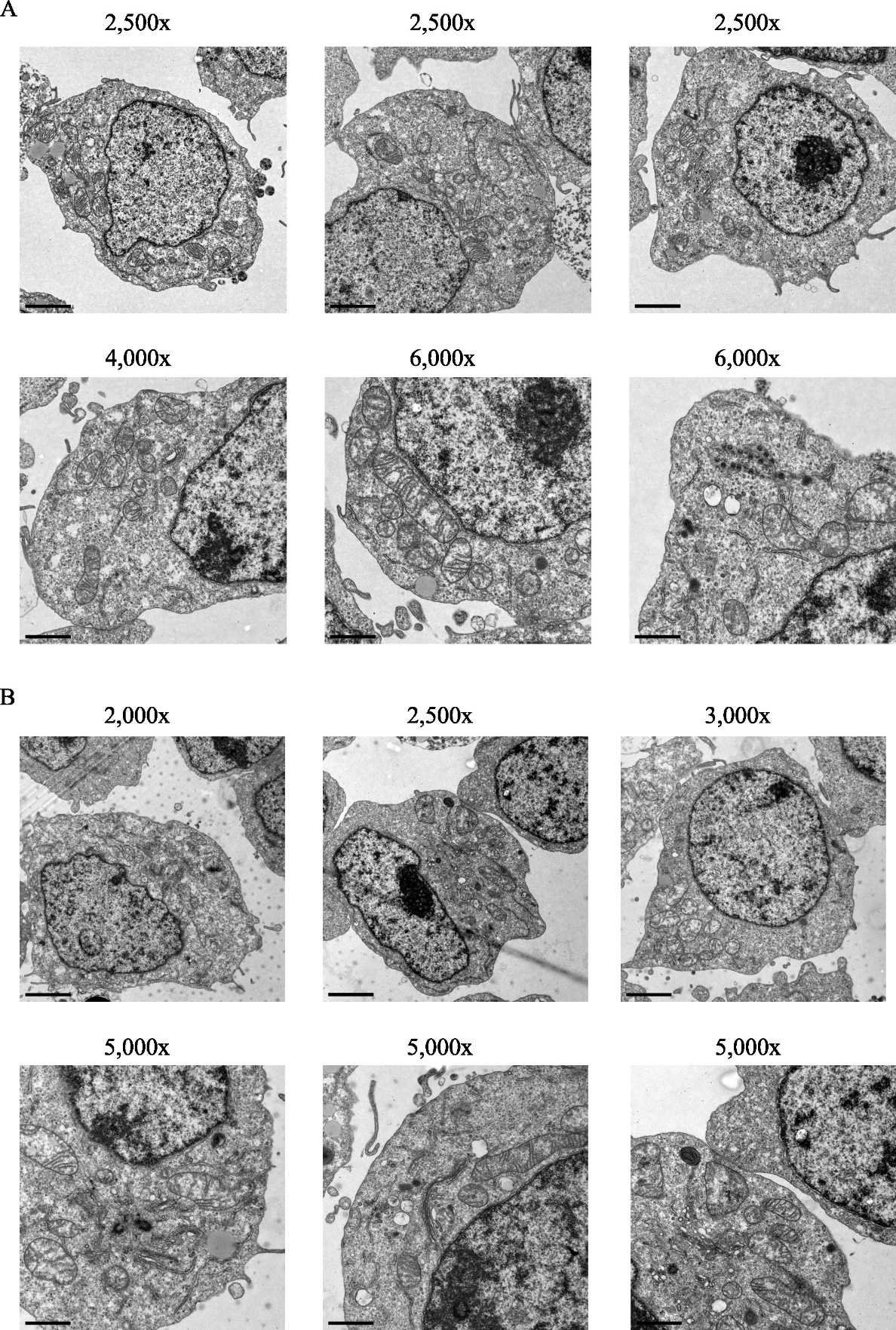


**Supplementary Figure 2.** EM images of nSMase2-APEX2 and H639A-APEX2 cells EM pictures of Jurkat cells expressing nSMase2-APEX2 (A) or H639A-APEX2 (B) fusion proteins taken at different magnifications.


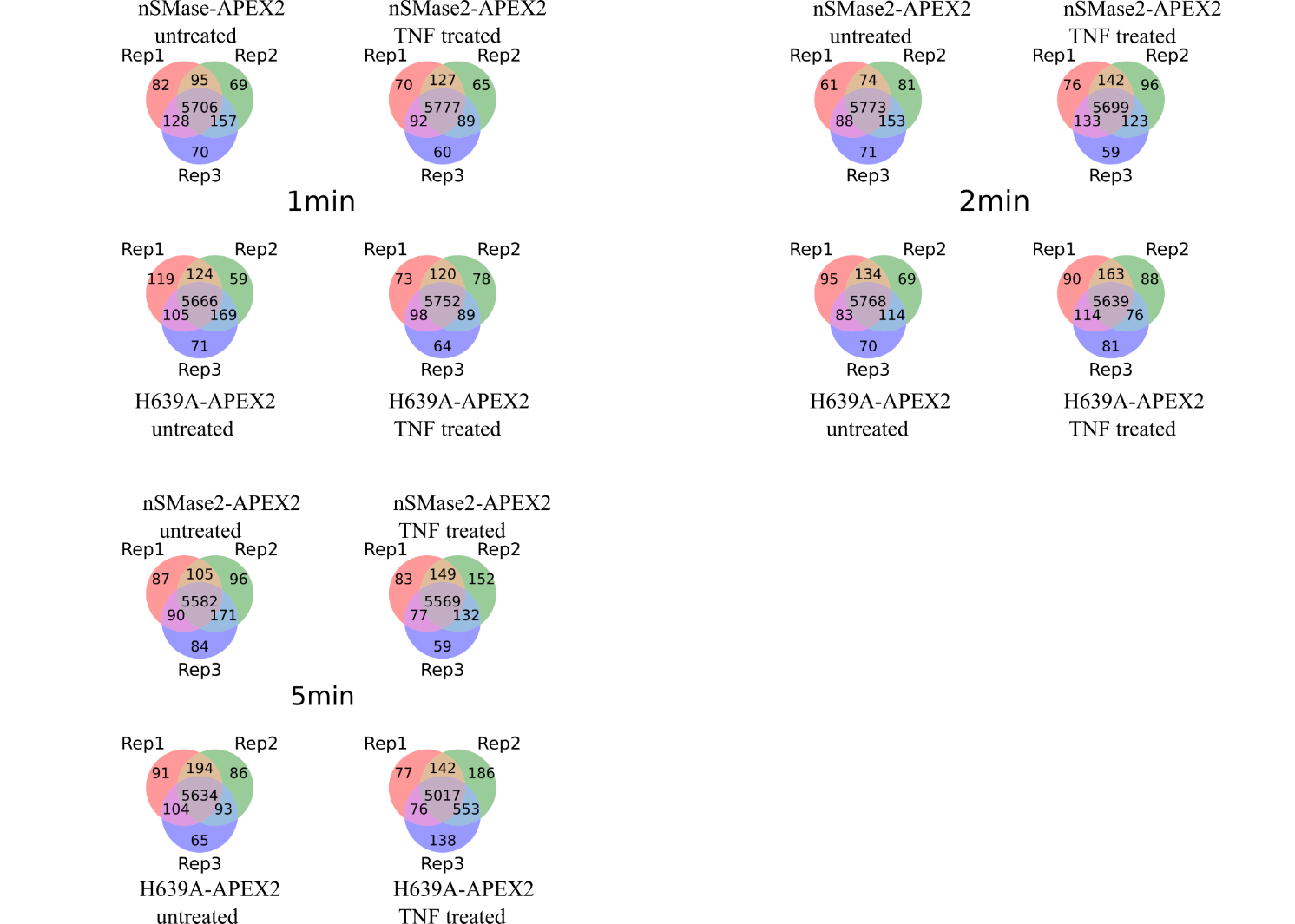


**Supplementary Figure 3.** Overlap of identified nSMase2 proximal proteins in three biological replicates. Venn diagrams showing the overlap of identified proteins of the three independent biological replicates fromAPEX2-mediated proximity labeling in TNFα-treated and non-treated nSMase2-APEX2 and H639A-APEX2 cells for all stimulation time points (1 min, 2 min, 5 min).


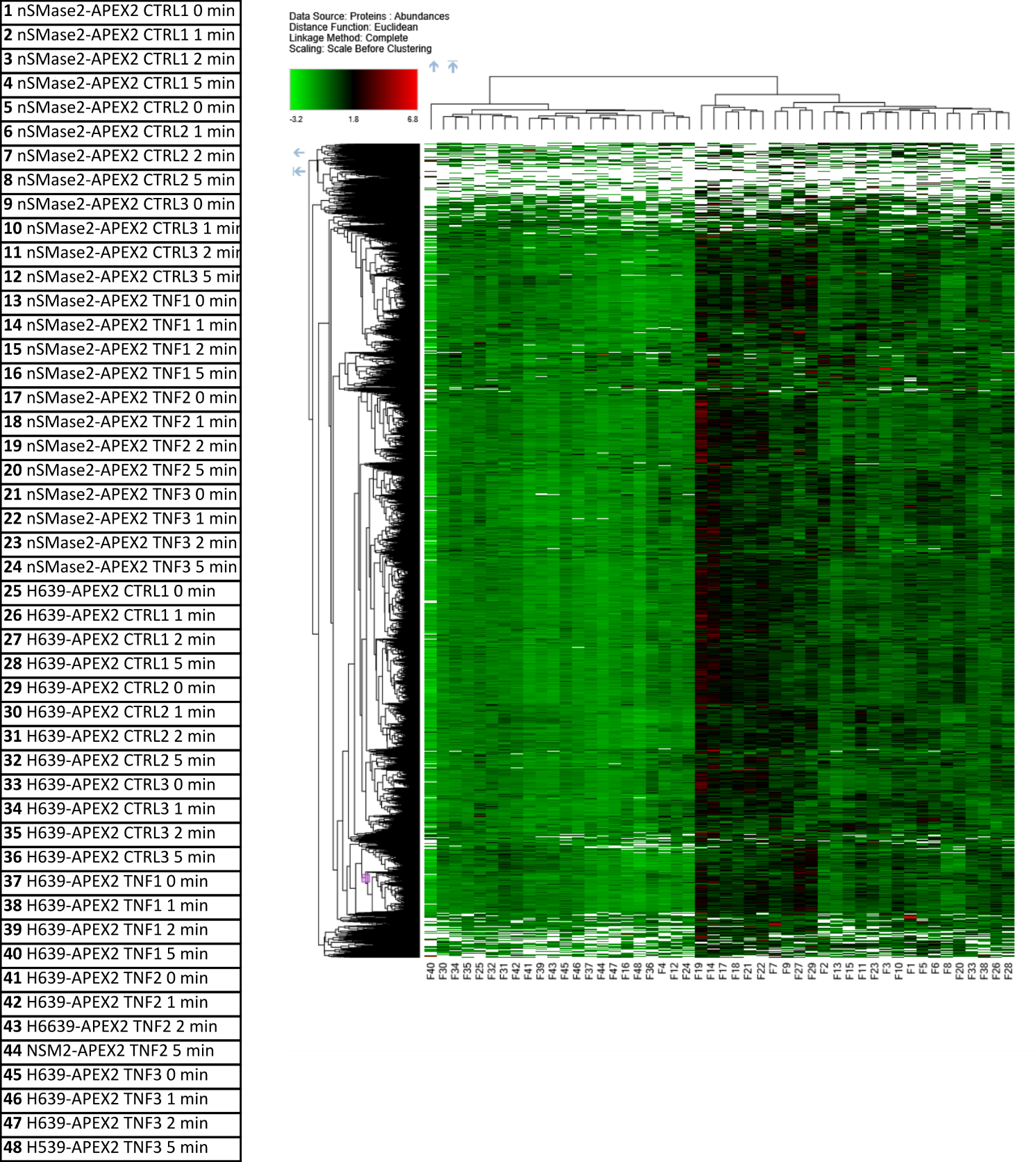


**Supplementary Figure 4.** Cluster map of protein abundances in the proximity labeling samples Cluster map of identified proteins showing protein abundance for each sample out of three independent biological replicates (n=3). Relative intensities for all proteins in each sample (measured as technical triplicates) are indicated by color-coding from green (< -3) to red (>6). Sample information (F1-F48) is provided in the table.


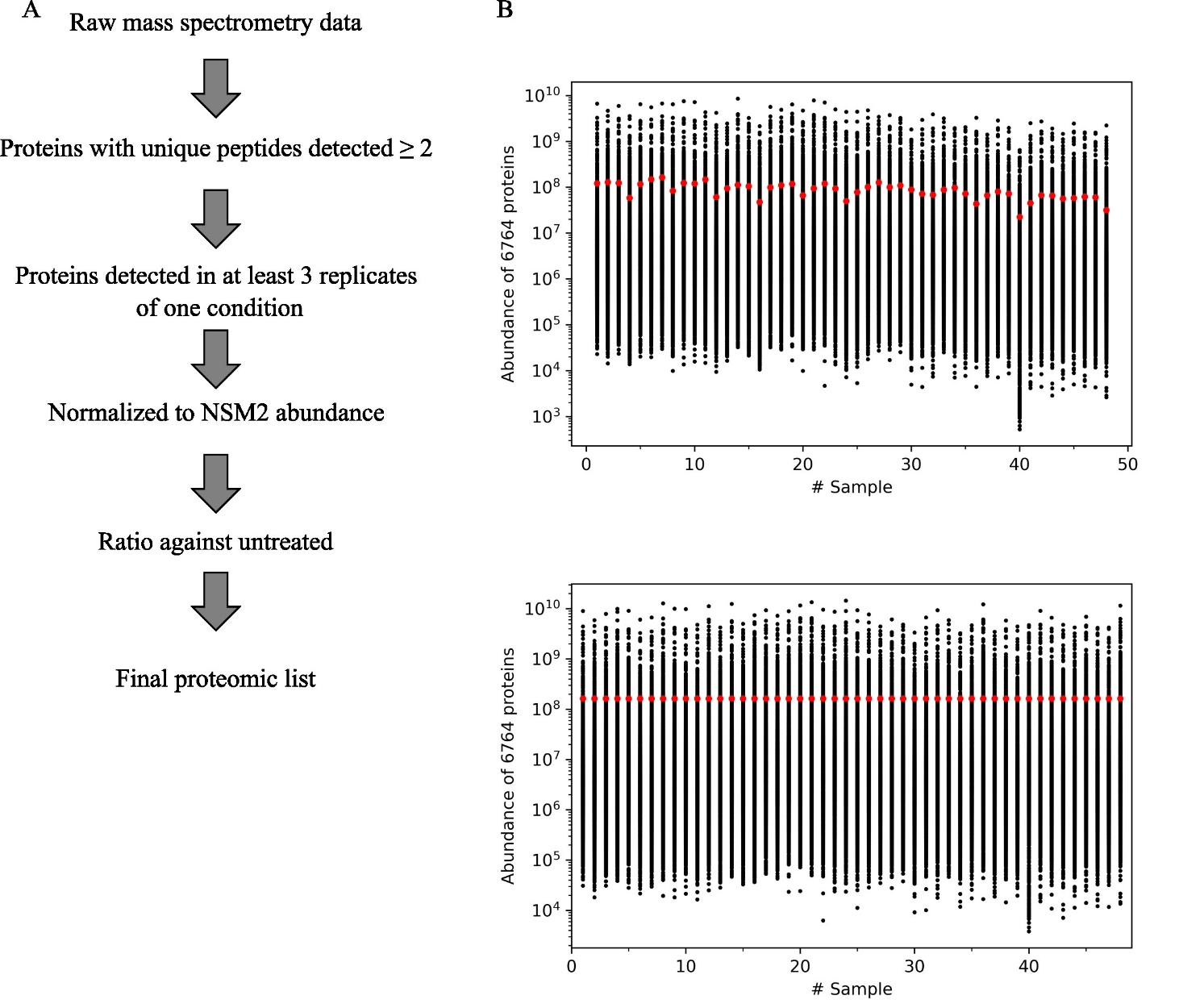


**Supplementary Figure 5.** Filtering approach. (A) Filtering approach used in this study for the identification of nSMase2 proximity proteins by mass spectrometry. (B) Protein abundancies in each sample out of three independent biological replicates (n=3) before, and after up-sampling normalization to nSMase2. nSMase2 values are highlighted in red in each sample (measured as technical triplicates).


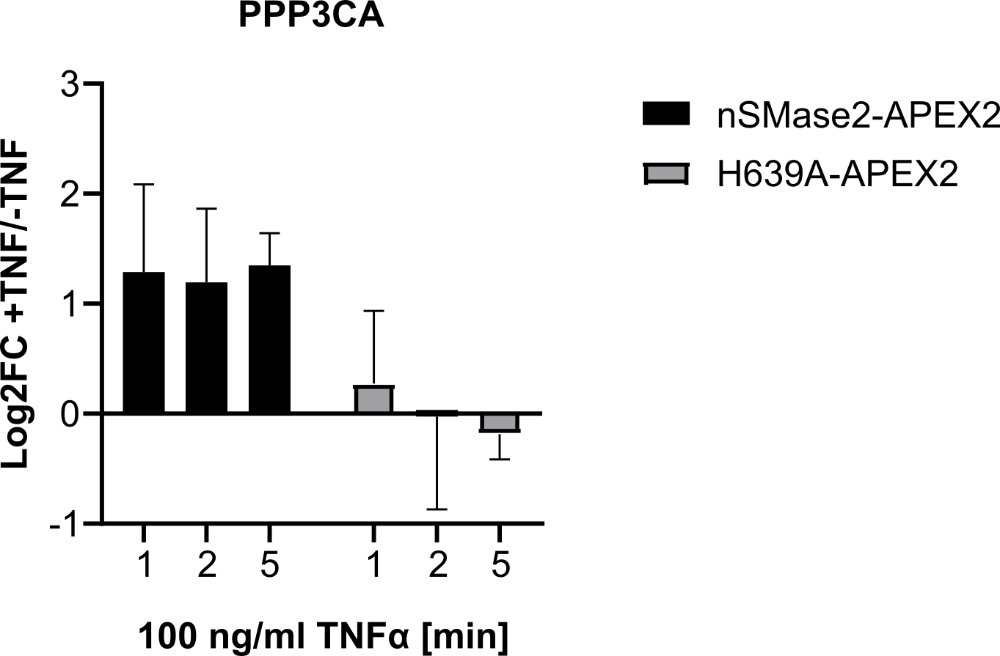


**Supplementary Figure 6.** Protein abundance and relative enrichment (Log2FC) of PPP3CA in nSMase2-APEX2 or H639A APEX2 after 1, 2, and 5 min of TNFα (100 ng/mL) stimulation compared to untreated nSMase2-APEX2 or H639A-APEX2 cells, respectively. Mean values of three independent experiments are presented with standard deviations.


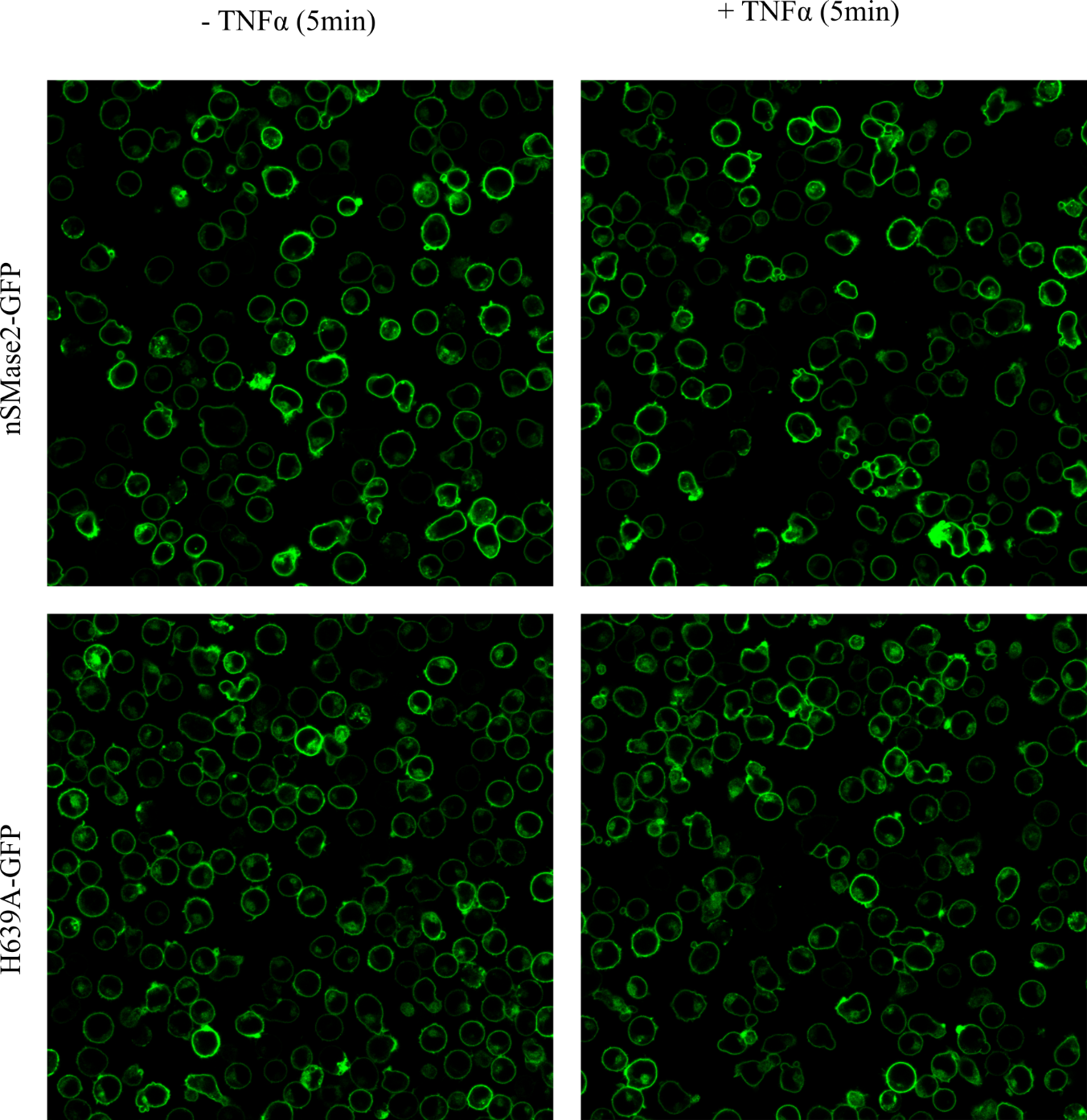


**Supplementary Figure 7.** nSMase2 localization in response to TNFα. Subcellular localization of nSMase2-GFP and H639A-GFP in Jurkat cells without and with 100 ng/mL TNFα stimulation for 5 min is visualized using confocal fluorescence microscopy.
