# Supplementary figures and images for: Dynamic changes in the proximitome of neutral sphingomyelinase-2 (nSMase2) in TNFα stimulated Jurkat cells
Source: Front Immunol. 2024 Jul 9;15:1435701. doi: 10.3389/fimmu.2024.1435701 (PMC11263205; doi:10.3389/fimmu.2024.1435701)

Figure 2B

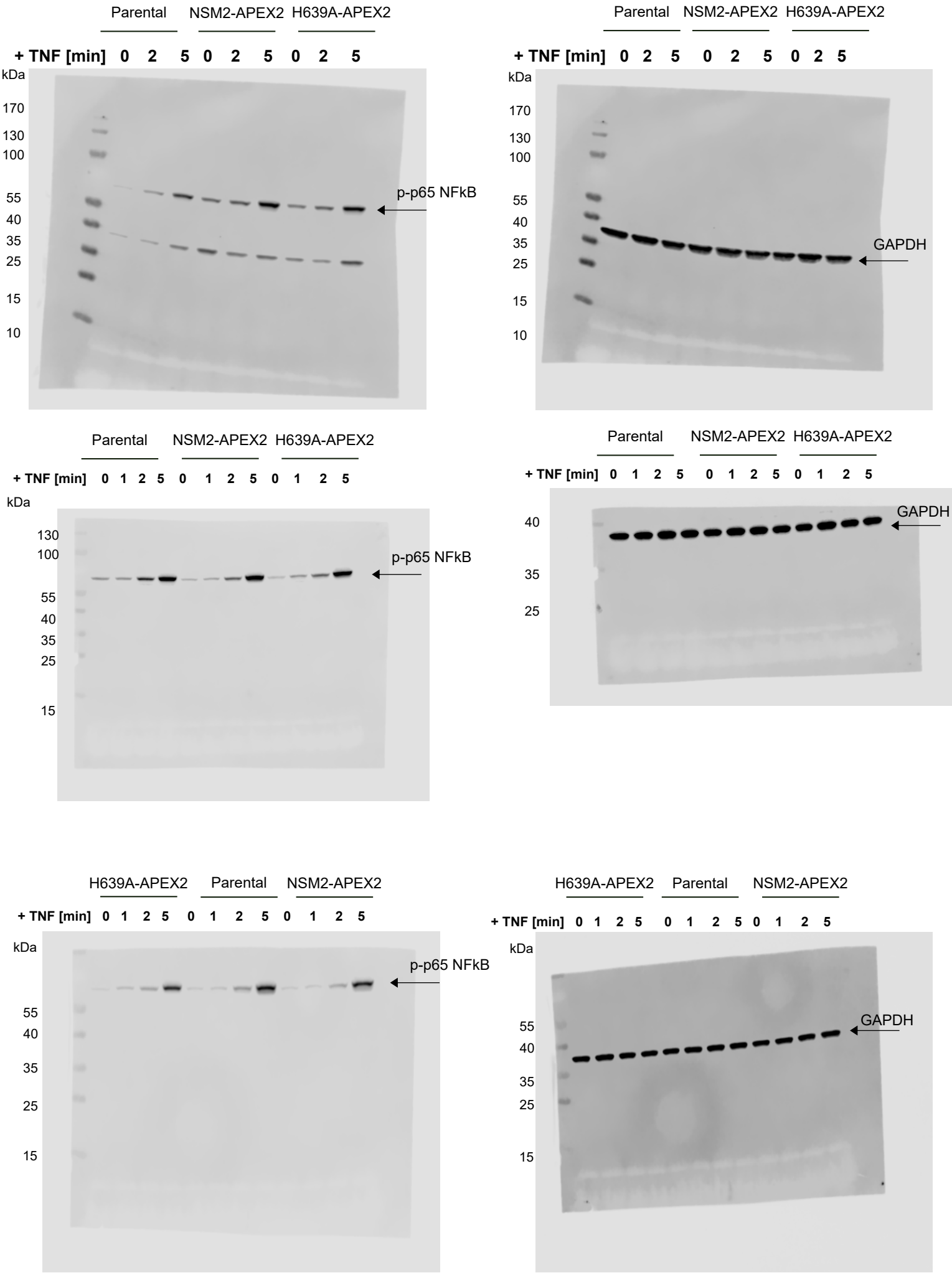

Figure 6 A

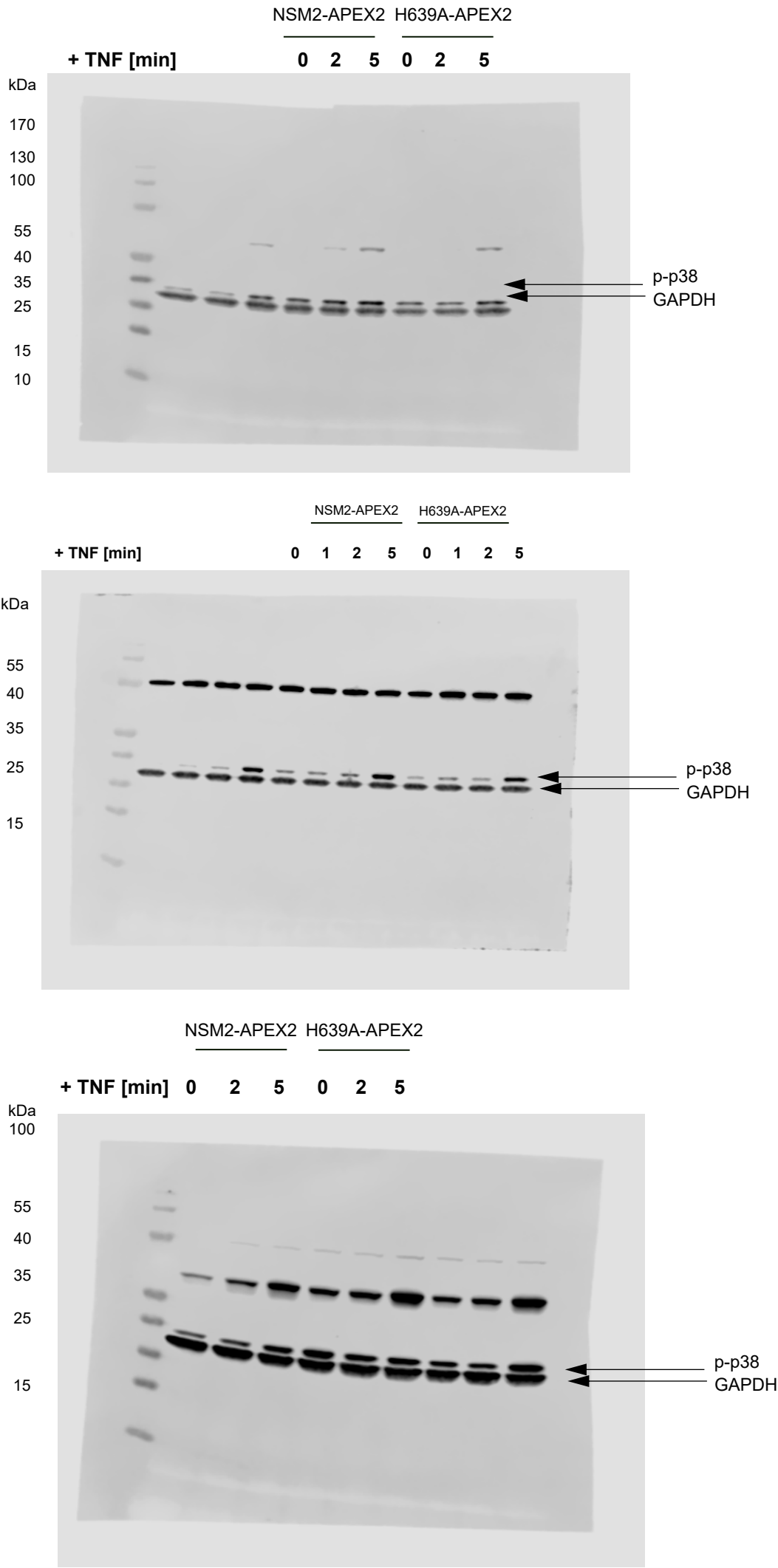

Figure 6 B

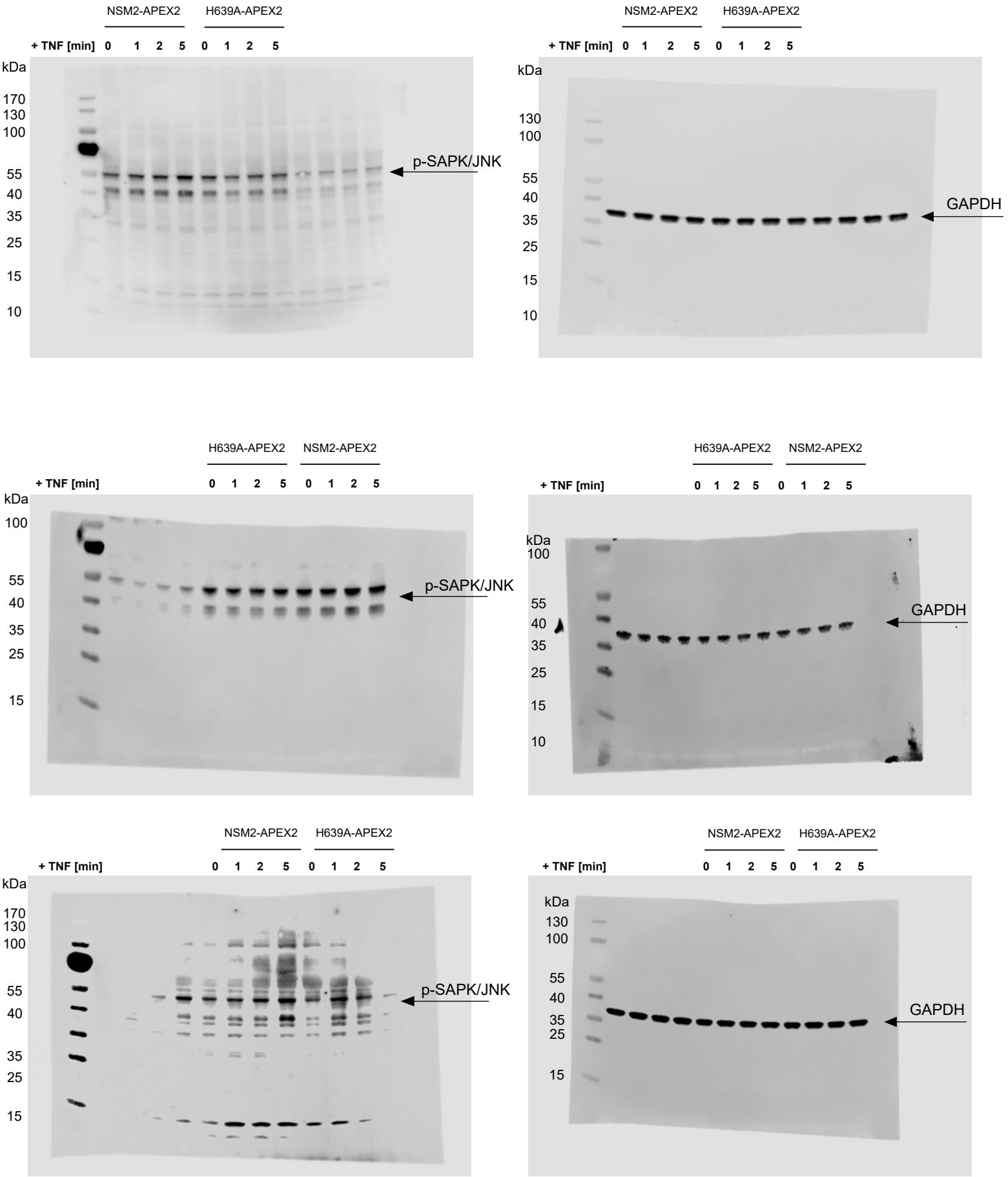

Figure 3 B

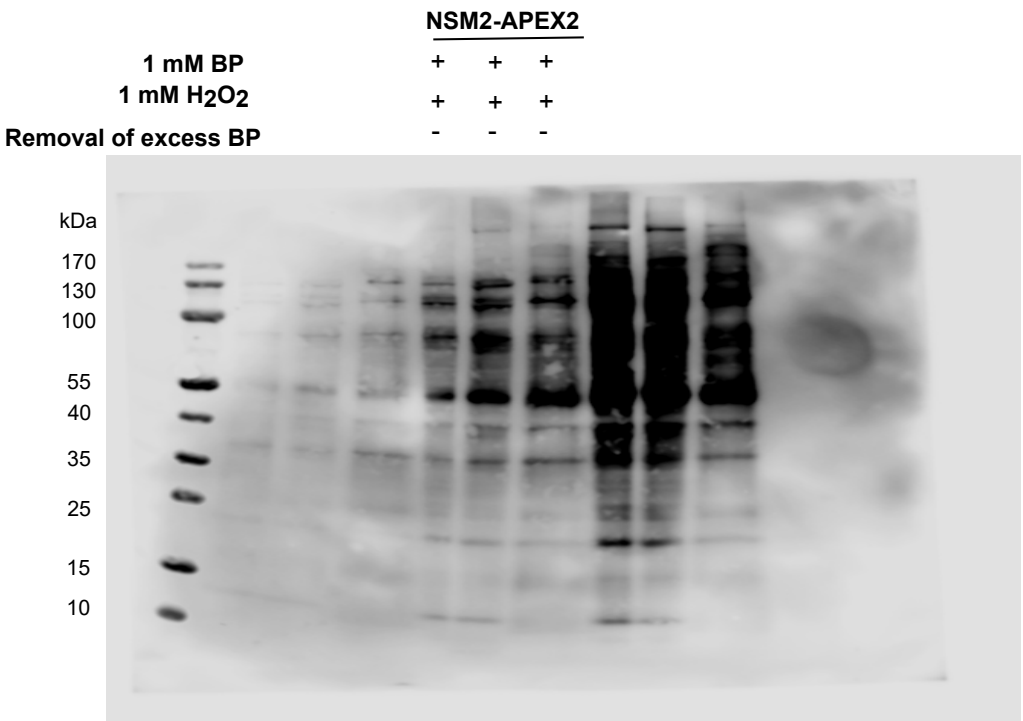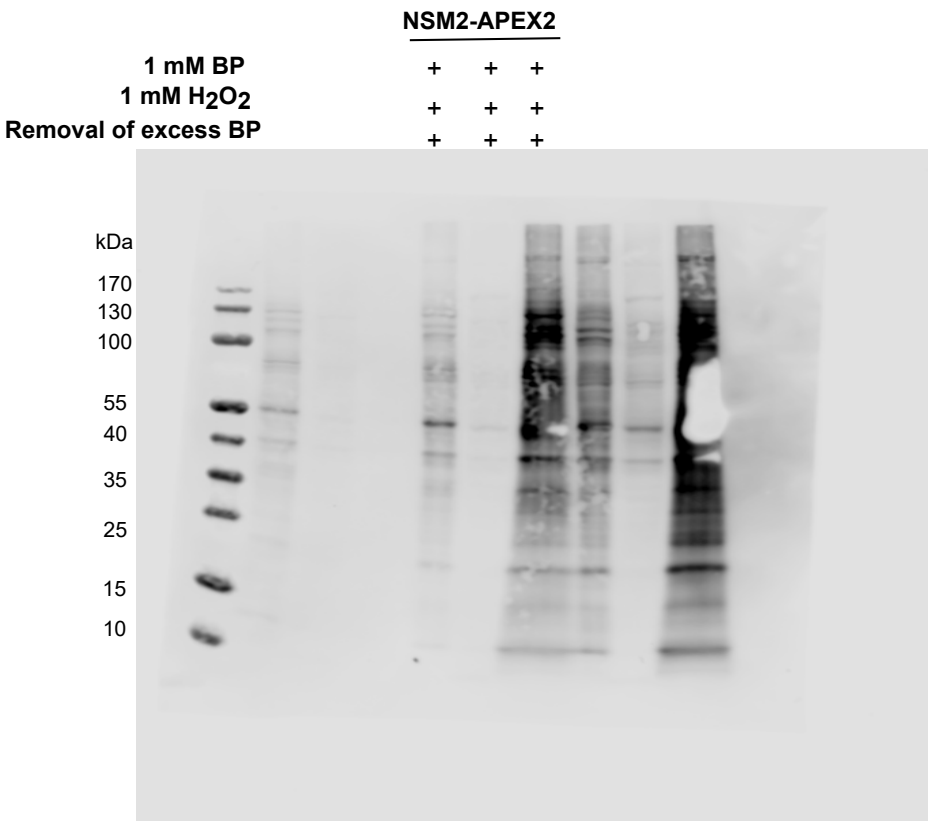

Figure S1B

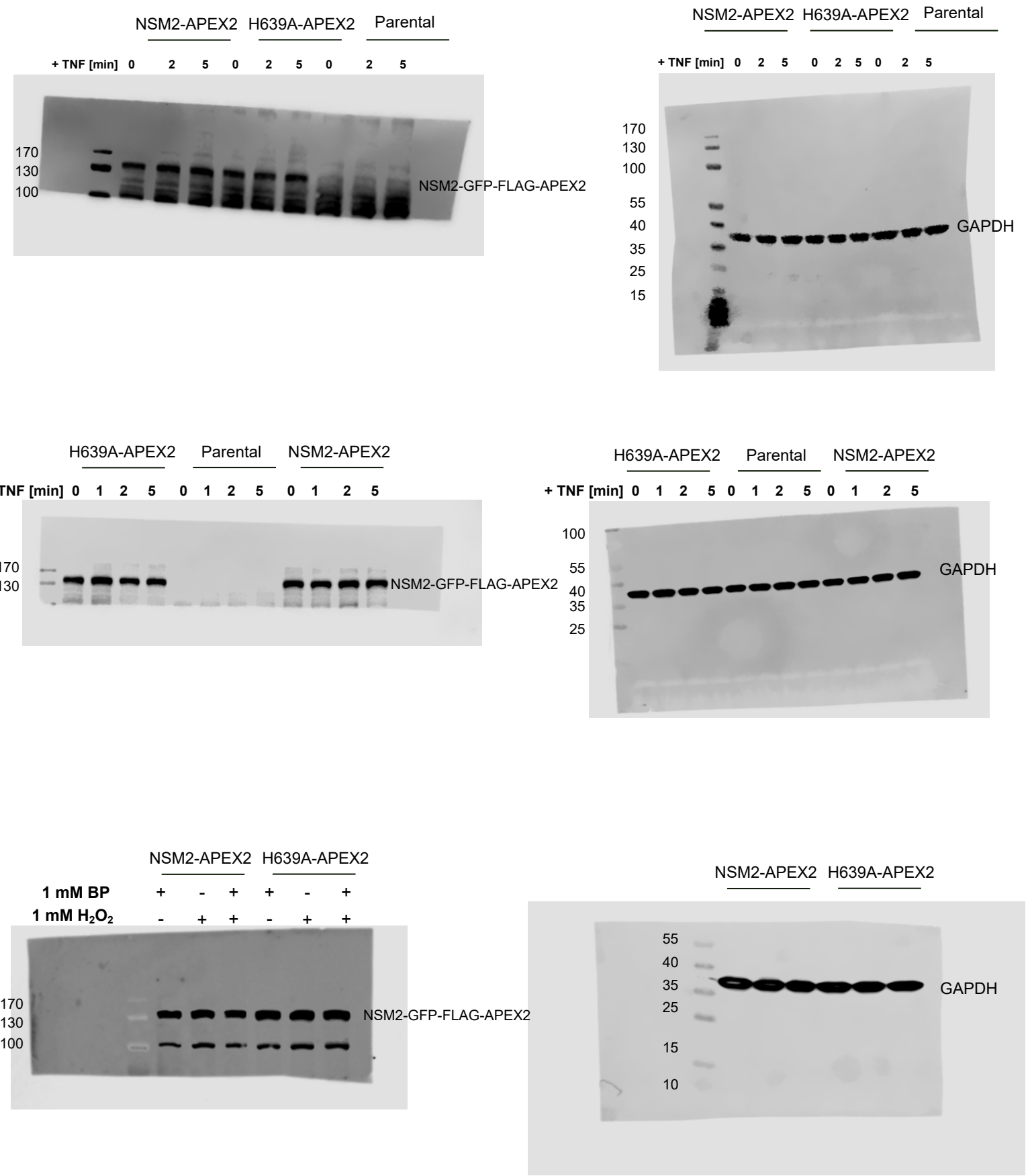

Supplement: Supplementary file 4 [file DataSheet_2.pdf]
